# Adsorption and Photocatalytic Activity of Covalent Organic Frameworks for Environmental Remediation: A Review of Density Functional Theory Studies

**DOI:** 10.3390/molecules31203695

**Published:** 2026-10-15

**Authors:** Damen Nurgaliyeva, Mirat Karibayev, Anuar Aldongarov, Aitkazy Kaisha, Zhaksylyk Suiindik, Bekzat Tynybekov, Aigerim Abeyeva, Zhambul Kerimkulov, Yerbolat Kalpakov, Raushan Baikanova, Ayazhan Igimbay, Galiya Baisalova, Sergei Piskunov

**Affiliations:** 1 Department of Natural Sciences, Astana International University, Astana 010000, Kazakhstan; damennurgalieva@gmail.com; 2 National Laboratory Astana, Nazarbayev University, Astana 010000, Kazakhstan; mirat.karibayev@nu.edu.kz (M.K.); aitkazy.kaisha@nu.edu.kz (A.K.); 3 Department of Chemistry, Lev Nikolayevich Gumilyov Eurasian National University, Astana 010000, Kazakhstan; enu-2010@yandex.kz; 4 Department of Chemistry, Nazarbayev University, Astana 010000, Kazakhstan; zhaksylyk.suiindik@nu.edu.kz; 5 Department of Botany and Agroecology, Al-Farabi Kazakh National University, Almaty 050000, Kazakhstan; 6 Department of Normal Physiology, National Medical University Named After Toregeldy Sharmanov, Astana 010000, Kazakhstan; 7 Department of Information Systems and Informatics, Abay Myrzakhmetov Kokshetau University, Kokshetau 020000, Kazakhstan; zhambul-k@yandex.ru; 8 School of Intelligent Systems, Astana IT University, EXPO C1, Mangilik El 55/11, Astana 010000, Kazakhstan; y.kalpakov@astanait.edu.kz; 9 School of Medicine, National Medical University Named After Toregeldy Sharmanov, Astana 010000, Kazakhstan; baikanova.r@amu.kz; 10 Department of Chemistry and Biotechnology, Shokan Ualikhanov Kokshetau University, Kokshetau 020000, Kazakhstan; tauzhanova97@mail.ru; 11 Institute of Solid State Physics, University of Latvia, LV-1063 Riga, Latvia

**Keywords:** water pollutants, adsorbents, covalent organic frameworks, purification, adsorption, photocatalysis, atomistic study

## Abstract

The contamination of water resources by heavy metals, radionuclides, and organic pollutants demands efficient materials for water treatment. Covalent organic frameworks (COFs) are promising candidates owing to their tunable porosity and functionalizable frameworks. This review examines density functional theory (DFT) studies of COFs for environmental remediation, distinguishing three frequently conflated processes: adsorptive capture, photocatalytic transformation, and combined adsorption–photocatalysis. Studies are analyzed across heavy metals, radionuclides, and organic pollutants, including antibiotics, pharmaceuticals, per- and polyfluoroalkyl substances (PFASs), dyes, pesticides, flame retardants, and others. Photocatalytic applications, including hydrogen peroxide (H_2_O_2_) production and others, are treated separately in terms of band alignment, charge separation, and reaction free energies. This review also assesses methodological limits of current DFT practice, including the incomparability of adsorption energies across functionals and model types, the distinction between adsorption energy and adsorption free energy, and the gap between computed energetics and experimental capacities.

## 1. Introduction

### 1.1. Covalent Organic Frameworks

**Figure 1:**
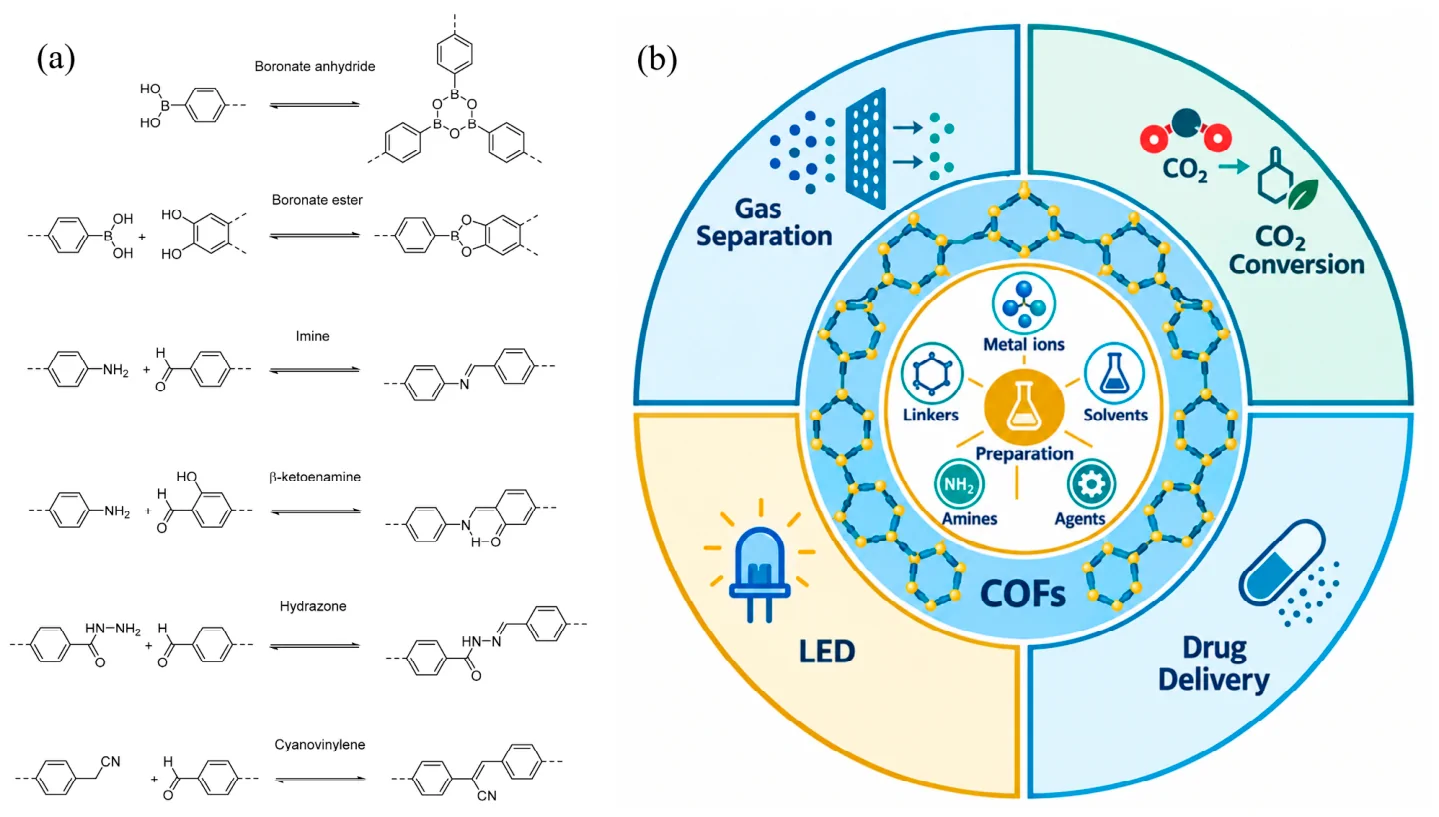
(**a**) Most used types of linkages in the construction of COFs. Reprinted with permission from (CC-BY license) [5]. (**b**) The overview of the preparation approaches of COFs and their applications. Reprinted with permission from (CC-BY license) [8].

Covalent organic frameworks (COFs) are crystalline porous materials constructed through the covalent assembly of organic building blocks via reversible condensation reactions (Figure 1a). Since their first report in 2005, COFs have attracted sustained interest because they combine permanent porosity, low density, and chemical and thermal stability with a structural precision that permits rational design of host–guest interactions [1,2,3,4,5]. Unlike metal–organic frameworks (MOFs), COFs are composed entirely of light elements (C, H, N, O, B, and S) linked through covalent bonds, which eliminates concerns about metal leaching and provides a chemically robust platform for water treatment. Their topology, pore size, and chemical functionality can be tuned through the choice of building blocks and linkage chemistry, enabling the deliberate incorporation of binding sites for target contaminants (Figure 1b) [5,6,7].

For the purposes of this review, the relevant properties of COFs are their chemical versatility and their structural tunability. Functionalization can be performed before framework formation, by modifying monomers, or after framework formation, by grafting thiol, carboxyl, amidoxime, or phosphoryl groups onto a pre-formed lattice. These functional groups determine whether a given COF captures a contaminant by electrostatic interaction, coordination, hydrogen bonding, or halogen bonding, and they also determine the material’s photocatalytic behavior when the framework is photoactive. The literature on COF synthesis, topology, and applications beyond environmental remediation is extensive and has been reviewed in many studies [9,10,11,12,13,14]. Herein, this review focuses on what density functional theory (DFT) calculations have established about how COFs interact with contaminants in water and on the methodological limitations (Section 2.3) that constrain the interpretation of those calculations.

Moreover, because COFs are explored both as adsorbents and photocatalysts, the conceptual boundaries between the processes are important to define. Herein, adsorption is the non-destructive retention of a contaminant at the COF surface or within its pores through non-covalent or coordinative interactions, governed by affinity, capacity, and selectivity; the pollutant’s chemical identity is unchanged. At the same time, photocatalytic transformation is a light-driven chemical process in which the COF generates electron–hole pairs and reactive oxygen species that cleave or rearrange chemical bonds, ideally mineralizing the contaminant; here, band gap, band alignment, and charge separation are the governing descriptors. Then, combined adsorption–photocatalysis refers to systems in which adsorption enriches the contaminant near photoactive sites and photocatalysis subsequently degrades it in situ, with the reaction regenerating free adsorption sites. 

### 1.2. Methodology of This Review (PRISMA Flow Diagram)

**Figure 2:**
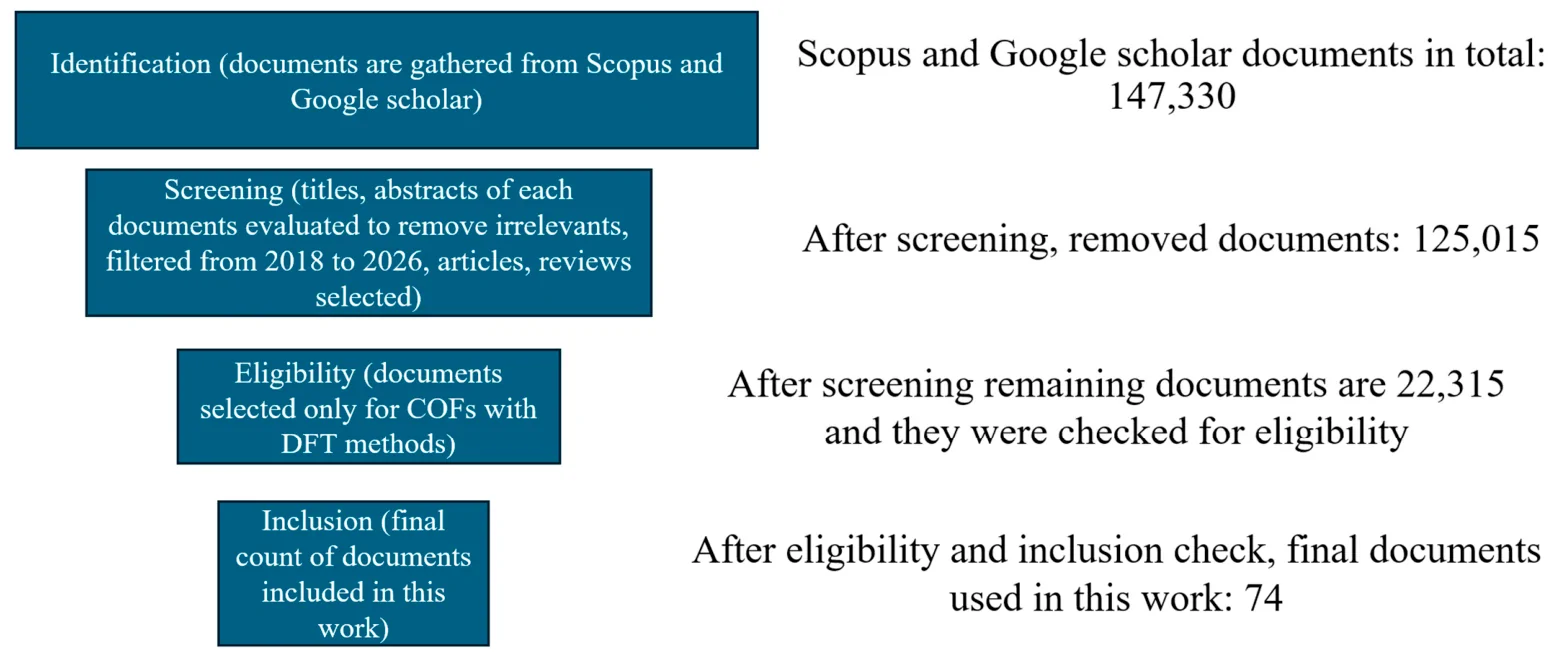
PRISMA flow diagram of this review.

This review was conducted according to a predefined search and screening protocol adapted from the Preferred Reporting Items for Systematic Reviews and Meta-Analyses (PRISMA) guidelines. The flow of records through identification, screening, eligibility, and inclusion is shown in Figure 2.

In the first step (identification), the literature was retrieved from Scopus and Google Scholar using the Boolean search string (“covalent organic framework” OR “COF” OR “COFs”) AND (“density functional theory” OR “DFT” OR “first-principles”) AND (“adsorption” OR “photocatalysis” OR “degradation” OR “removal” OR “capture”) AND (“water” OR “wastewater” OR “environmental” OR “remediation” OR “pollutant”). The initial search returned 147,330 records across the two databases, a total that reflects the breadth of the search string and the inclusion of Google Scholar.

In the second step (screening), documents were screened by title and abstract to remove duplicates, non-English sources, conference abstracts, book chapters, preprints, and studies unrelated to COF-based environmental remediation. Records were further filtered to the period 2018–2026 and restricted to peer-reviewed articles and reviews, reflecting the fact that DFT studies of COF adsorption and photocatalysis only became a substantial body of the literature after 2018. This stage removed 125,015 records, leaving 22,315 for eligibility assessment.

Regarding the third step (eligibility), the remaining 22,315 records were assessed in full text against the inclusion criteria: (i) the study reports first-principles DFT calculations of contaminant adsorption, photocatalytic transformation, or combined adsorption–photocatalysis on COF-based materials; (ii) the target application is water or wastewater treatment; (iii) at least one of the following quantities is reported: adsorption energy, adsorption free energy, binding geometry, charge-transfer magnitude, band structure, or reaction free energy. Records were excluded if they employed only force-field, semi-empirical, or classical molecular dynamics methods without a DFT component.

After eligibility assessment, 74 studies met all inclusion criteria and were retained for the qualitative synthesis and, where data permitted, for the discussions presented in Section 3, Section 4, Section 5 and Section 6. These 74 studies are distributed across heavy metal adsorption (Section 3), radionuclide adsorption (Section 4), organic pollutant adsorption (Section 5), and photocatalytic transformation (Section 6). 

### 1.3. Scope and Organization of This Review

This review examines DFT studies of COF-based materials for environmental remediation. Specifically, we address four questions: (i) what DFT calculations have established for the removal of heavy metals, radionuclides, and organic pollutants by COFs through adsorption or photocatalytic activity; (ii) how adsorptive capture and photocatalytic transformation are mechanistically distinguished, and where the two processes couple; (iii) how functional choice, dispersion treatment, basis set, relativistic treatment, and periodic-versus-cluster modeling affect the reported quantum-chemical properties; (iv) how DFT-computed properties relate to experimentally measured quantities.

This review is organized as follows. Section 2 presents the principles and applications of DFT, covering its theoretical foundations, its use in COF characterization and design, and the critical methodological considerations and limitations that govern the interpretation of computed adsorption energies and photocatalytic descriptors. Section 3, Section 4 and Section 5 survey DFT studies, in parallel with the corresponding experimental work, on the removal of heavy metals, radionuclides, and organic pollutants by COFs through adsorption. Section 6 addresses photocatalytic transformation as a mechanistically distinct process. Section 7 presents challenges and future perspectives.

## 2. Density Functional Theory: Principles and Applications

### 2.1. Theoretical Foundations of Density Functional Theory

DFT has emerged as the most widely employed electronic structure method in computational chemistry and materials science, offering a compelling balance between computational efficiency and predictive accuracy for systems of chemical and technological relevance. The theoretical foundation of DFT rests upon the Hohenberg–Kohn theorems, which establish that the ground-state electronic energy of a many-electron system is uniquely determined by its electron density distribution rather than the computationally intractable many-electron wavefunction. This profound insight reduces a 3N-dimensional problem to a three-dimensional one, enabling the treatment of systems comprising hundreds of atoms.

The practical implementation of DFT relies on the Kohn–Sham approach, which maps the interacting electron system onto a fictitious non-interacting system moving in an effective potential. The total energy is expressed as a functional of the electron density:

$$E\left[\rho \right]=T_{s}\left[\rho \right]+E_{ext}\left[\rho \right]+E_{H}\left[\rho \right]+E_{xc}\left[\rho \right]$$

where ρ denotes the electron density, E[ρ] is the total energy, $T_{s}[\rho ]$ represents the kinetic energy of non-interacting electrons, $E_{ext}[\rho ]$ accounts for electron–nucleus interactions, $E_{H}[\rho ]$ describes classical Coulomb interactions (Hartree energy), and $E_{xc}[\rho ]$ encapsulates all many-body exchange-correlation effects. The exchange-correlation functional, which remains unknown exactly, is the central challenge in DFT and must be approximated through various levels of theory [11,12,13].

The accuracy of DFT calculations critically depends on the chosen exchange-correlation functional. Local density approximation (LDA) assumes that the exchange-correlation energy at each point depends only on the local electron density. Generalized gradient approximation (GGA) functionals, such as Perdew–Burke–Ernzerhof (PBE), incorporate density gradients, significantly improving predictions for molecular geometries and adsorption energies. Meta-GGA functionals include kinetic energy density as an additional variable, while hybrid functionals incorporate a fraction of exact Hartree–Fock exchange, substantially enhancing accuracy for electronic properties such as band gaps and ionization potentials [14,15,16,17]. However, standard DFT functionals often fail to adequately describe weak intermolecular interactions such as van der Waals forces, which are crucial for modeling adsorption phenomena. This limitation is addressed through empirical dispersion corrections, including DFT-D3 and DFT-D4 schemes, which add an atom-pairwise energy term to account for long-range correlation effects.

### 2.2. DFT Applications in COF Characterization and Design

DFT calculations have proven indispensable for elucidating the structural, electronic, and adsorption properties of COF-based materials. At the structural level, DFT geometry optimization enables prediction of equilibrium framework conformations, including layer stacking arrangements in 2D COFs and pore dimensions. These calculations are critical for interpreting experimental powder X-ray diffraction patterns and validating proposed structural models. For adsorption studies, DFT provides atomic-level insights into binding energies, preferential adsorption sites, charge transfer pathways, and interaction mechanisms that are often inaccessible through experimental techniques alone [15,16,17,18,19,20,21].

Electronic structure analysis through DFT enables characterization of band structures, density of states (DOS), and frontier molecular orbitals, which are fundamental for understanding photocatalytic activity, electrical conductivity, and optical properties of COFs. The calculation of projected density of states (PDOS) identifies specific atomic orbital contributions to valence and conduction bands, guiding the rational design of photocatalysts with optimized band alignments for targeted redox reactions. Furthermore, electrostatic potential (ESP) mapping reveals regions of charge accumulation and depletion, enabling prediction of favorable binding sites for charged and polar contaminants. The integration of DFT calculations with experimental characterization has become a standard paradigm in COF research, enabling structure–property correlation and guiding the rational development of high-performance materials for environmental remediation.

Moreover, it is important to distinguish between adsorption energy and adsorption free energy when interpreting DFT results. The adsorption energy (*E_ad_*_s_), typically computed at 0 K, represents the electronic binding strength between an adsorbate and the COF framework and is widely used to compare relative affinities, identify preferential adsorption sites, and assess interaction mechanisms. However, because most DFT calculations neglect thermal, entropic, and solvation contributions, *E_ad_*_s_ should not be directly equated with the adsorption free energy (Δ*G_ads_*) that governs adsorption under experimental conditions, particularly in aqueous systems where solvent effects, temperature, and entropy play significant roles. Where statements concerning spontaneity, thermodynamic stability, or experimentally observed adsorption behavior are made, Δ*G_ads_* should be reported, computed by combining DFT electronic energies with zero-point energy (ZPE), thermal corrections, entropic contributions, and explicit or implicit solvation models.

### 2.3. Critical Methodological Considerations and Limitations for DFT Studies of COF Adsorption and Photocatalysis Activities

The DFT studies reviewed in this work provide atomic-level mechanistic insight that is difficult to obtain experimentally, but their quantitative interpretation is subject to several systematic limitations. 

The first limitation is functional dependence and the incomparability of adsorption energies. The exchange-correlation functional is the single largest source of discrepancy between reported adsorption energies. GGA functionals such as PBE and PW91 systematically underestimate charge-transfer and covalent binding by 0.2–0.5 eV relative to hybrid functionals, while hybrid functionals such as HSE06, PBE0, and B3LYP recover a portion of exact exchange and typically strengthen binding by 10–30% for chemisorbed ionic species. Meta-GGA functionals including M06-L, M06-2X, and SCAN occupy an intermediate position but were parameterized on different training sets and can differ from one another by 0.1–0.3 eV. In practice, HSE06 and PBE0 are used predominantly for periodic electronic-structure calculations of band gaps and DOS, while B3LYP and M06-2X dominate molecular cluster models of localized binding sites. The choice of functional can therefore shift the computed adsorption energy by 0.3–1.0 eV for the same physical system.

The second important part is the dispersion treatment. For physisorbed organic pollutants dominated by π–π stacking, van der Waals forces, and hydrophobic association, the inclusion or omission of an empirical dispersion correction changes the computed adsorption energy by 20–100 kJ/mol, which is often larger than the binding energy itself. The reviewed literature employs DFT-D2, DFT-D3, DFT-D3(BJ), DFT-D4, and, in some cases, no dispersion correction at all, and the choice is frequently not justified. Herein, the absence of dispersion correction can qualitatively change the predicted binding mechanism, converting what is in reality physisorption into an apparent weak chemisorption or, conversely, predicting no binding where dispersion-corrected calculations find a stable complex. 

Thirdly, solvation and pH-dependent speciation are important. Most of the reviewed calculations employ implicit solvent models, including PCM, SMD, or COSMO, which represent water as a structureless dielectric continuum. This approximation captures the leading electrostatic screening effect but omits the explicit first solvation shell, hydrogen bonding to framework functional groups, specific ion pairing with the adsorbate, and the structured water layer that forms at the hydrophobic and hydrophilic domains of the COF surface. The omission is particularly consequential for heavy-metal and radionuclide adsorbates, for which their aqueous speciation depends strongly on pH and solution composition. 

Fourthly, another important part is the idealization of defect-free framework structures. DFT calculations assume periodic, defect-free COF lattices with a single, well-defined stacking arrangement. Experimental COFs contain missing-linker and missing-node defects, grain boundaries between crystalline domains, stacking faults, and variable interlayer registry. Defect sites frequently exhibit different local chemistry, undercoordinated metal-binding atoms, exposed edges, and altered pore environments, and they can dominate adsorption at low contaminant concentrations. Because defects are irregular by definition, they are inaccessible to standard periodic DFT without explicit defect models, and no reviewed study explicitly parameterizes defect density.

A fifth important point to consider is the neglect of dynamic framework behavior. Static DFT calculations optimize a single minimum-energy geometry at 0 K. Real COFs exhibit linker rotation, framework flexibility, and, in some cases, pore-breathing transitions that alter the accessibility and binding geometry of adsorption sites at experimental temperatures. The dynamic response of the framework to the adsorbate includes local relaxation, hydrogen-bond rearrangement, and, in the most flexible frameworks, a genuine gate-opening transition, which is not captured by a single-point geometry optimization. Ab initio molecular dynamics and metadynamics can address these effects but remain computationally prohibitive for the system sizes considered in most reviewed studies, and only a small minority of the surveyed works employ them.

Moreover, it is important to note whether a periodic or cluster approach is used. Cluster models (finite molecular fragments treated in Gaussian or ORCA) truncate the COF at the edges of the model, artificially removing conjugation, interlayer interactions, and long-range electrostatics. Periodic models (VASP, CASTEP, and DMol3 with periodic boundary conditions) retain these effects but introduce their own artifacts, including the choice of unit cell and k-point sampling, the fixed AA or AB stacking assumed for two-dimensional COFs, and the treatment of vacuum spacing between layers. For the same COF–adsorbate pair, cluster and periodic calculations frequently differ by 0.3–0.8 eV and can predict different preferred binding sites. In this regard, periodic models are generally preferable for extended two-dimensional COFs and for interlayer adsorption, while cluster models remain appropriate for localized binding-site analysis and for hybrid-functional calculations where periodic hybrid DFT is prohibitively expensive.

Also, it is important to note the quantitative comparison between 0 K energetics and ambient-condition capacities. The most persistent limitation is the one already introduced above. DFT reports an electronic adsorption energy at 0 K in vacuum or in an implicit continuum, whereas experimental capacities are measured at ambient temperature in aqueous solutions at a defined pH, with competing ions, surface protonation equilibria, and mass-transport limitations. The gap between the two quantities is not a single correction but a sum of contributions: thermal and zero-point energy, the entropy penalty of adsorption, the free energy of desolvation, pH-dependent speciation of the adsorbate, competitive occupation of binding sites by various matrix ions, and diffusion limitations in porous frameworks. 

Finally, basis set dependence and pseudopotential/relativistic treatment are important to note. Basis set incompleteness and inconsistent pseudopotentials can shift adsorption energies by 0.1–0.5 eV, while relativistic effects become significant for heavy-metal and radionuclide adsorbates.

## 3. DFT Studies on Heavy Metal Adsorption

This section reviews DFT investigations of heavy metal adsorption on COFs [22,23,24,25,26,27,28,29,30,31,32,33,34,35,36,37,38], with attention to three questions: (i) why reported adsorption energies for chemically similar systems differ by up to an order of magnitude; (ii) how much of this spread originates from methodological choices (functional, dispersion correction, model type, and solvation); and (iii) to what extent DFT-computed binding energies can be quantitatively mapped onto experimentally measured adsorption capacities.

Herein, most reviewed calculations report $E_{\mathrm{ads}}$, an electronic energy computed at 0 K for a fixed geometry, often in vacuum or an implicit continuum. This quantity is not the one that governs adsorption under experimental conditions, nor is it directly proportional to the Langmuir or Sips saturation capacity ($q_{\max}$). Where reported by the original authors, we retain their notation and explicitly state what is reported.

### 3.1. Mercury (Hg^2+^)

Mercury adsorption has been studied on boroxine COFs, Ag nanoparticle–COF composites, thiourea- and thiol-functionalized COFs, and magnetic COF–ligand hybrids. Li and Zhang [22] used HSE06/6-311+g(d) with LANL2DZ for Hg and a PCM solvent model to examine Hg^2+^ on pristine boroxine COF. They reported $E_{\mathrm{binding}}=-32.14$ kcal mol^−1^, an interaction distance of 2.43 Å, and charge transfer of 0.53 e from the boroxine layer to Hg^2+^, with a decrease in the HOMO-LUMO gap [22]. The ionic character of the interaction and the magnitude of the gap reduction are plausible. However, the absence of an explicit dispersion correction and the use of a cluster model limit comparability with periodic calculations on extended COFs.

Wang et al. [23] combined experiments with DFT for Ag NPs anchored on COF-LZU1. Using PW91/BS-II and M06-2X/BS-I with NBO analysis, they found Hg adsorption on ($E_{\mathrm{ads}}$) Ag top sites at −379.50 kcal mol^−1^ (PW91) and −325.22 kcal mol^−1^ (M06-2X) versus −230.20 to −234.72 kcal mol^−1^ on pristine COF-LZU1 [23]. The ~54 kcal mol^−1^ difference between PW91 and M06-2X for the same site is a direct illustration of functional dependence: GGA functionals such as PW91 systematically overestimate binding for strongly interacting metal–metal systems, whereas meta-hybrids such as M06-2X partially correct this. The experimental $q_{\max}$ was 113 mg g^−1^ with 150% Ag atom utilization, and the formation of Ag-Hg amalgam was confirmed by XRD and HR-TEM. The mechanistic conclusion is reduction and amalgamation, and the absolute energies are not transferable across functionals. 

Li et al. (2025) [24] synthesized a S,N-rich COF (COF_ETTA-BPT-S,N) via thiol–yne click chemistry and performed B3LYP/SDD calculations with MBO and IRI analysis for three Hg(II) species: Hg^2+^, Hg(OH)^+^, and Hg(OH)_2_ [24]. MBO analysis gave 0.5025 for Hg–S and 0.4310 for Hg–N (most stable configurations). Next, IRI confirmed that Hg(OH)^+^ binds via coordination, while Hg^2+^ and Hg(OH)_2_ interact through notable attraction and van der Waals forces. Experimentally, the material reduced 30 μg L^−1^ Hg(II) to 0.28 μg L^−1^ within 10 s (meeting the WHO drinking-water limit) and reached $q_{\max}=621.65$ mg g^−1^. This study is notable because the authors explicitly modeled pH-dependent speciation, which is a practice that should be standard but is rare in the reviewed literature.

Yang et al. (2022) [25] prepared a magnetic COF loaded with a Schiff-base ligand (MCM). M06-2X/6-31G(d,p) with LANL2DZ was implemented to study the binding energy for the Schiff-base–Hg complex and ligand–COF binding [25]. The experimental $q_{\max}$ was 843.89 mg g^−1^ with a detection limit of 0.01 μg L^−1^. The use of M06-2X, a meta-hybrid parameterized partly for non-covalent interactions, is more defensible than pure GGA for this system. 

In general, the varying values of $E_{\mathrm{binding}}$ and $E_{\mathrm{abs}}$ are due to different dominant contributors, which are as follows: (i) the functional (GGA vs. meta-hybrid vs. hybrid); (ii) whether the adsorbate is the bare ion or a pH-relevant hydroxo species; (iii) whether the model is a finite cluster or a periodic slab; and (iv) whether dispersion is included.

### 3.2. Lead (Pb^2+^)

Li and Zhang (2021) [22] reported Pb^2+^@boroxine as the strongest of the three cations studied, with $E_{\mathrm{binding}}=-70.81$, an interaction distance of 2.05 Å, charge transfer of 0.91 e, a HOMO–LUMO gap of 0.73 eV (86% reduction), and an electron-transfer rate of 1.84 × 10^15^ s^−1^ [22]. TD-DFT showed ligand-to-metal charge-transfer excitations with a red shift. The trend Pb^2+^ > Hg^2+^ > Cd^2+^ is physically reasonable on ionic-radius and HSAB grounds.

Xiang et al. (2024) [26] designed a thiadiazole-functionalized COF (COF@HPLC) and used B3LYP/SDD with ESP mapping and five adsorption models [26]. The N3 site gave the shortest Pb–N distance and exhibited the strongest binding; the S2 site exhibited the weakest binding. Experimentally, $q_{\max}=251.37$ mg g^−1^ at pH 4, with pseudo-second-order kinetics and Langmuir isotherm behavior. The agreement between DFT site preference and XPS shifts (N 1s, S 2p) is a good example of theory–experiment cross-validation.

Saad et al. (2022) [29] examined Pb^2+^ complexation with CN-functionalized biphenyl boroxine COF (PBCOF-CN) at ligand:metal ratios from 1:1 to 1:4 [29]. Pb–ligand distances (2.161–3.372 Å) were shorter than those for Hg (3.575–3.748 Å) or Cd (3.404–3.428 Å), consistent with Pb^2+^’s smaller effective ionic radius in this coordination environment. Natural population analysis gave a Pb charge of 0.411, indicating electron donation from the cyano group. TD-DFT predicted a red shift to 510 nm for Pb^2+^@PBCOF-CN, the largest among the studied metals. This study is unusual in reporting a metal-to-ligand ratio series, which is more realistic than a single 1:1 model.

The difference in their energies and other quantum chemical property values is partly physical (different COF chemistry) and partly methodological (HSE06 with exact exchange strengthens ionic binding relative to B3LYP). A quantitative ranking of Pb^2+^ affinity across COFs would require a single functional, a single model type, and a consistent solvation treatment.

### 3.3. Cadmium (Cd^2+^)

Cd^2+^ is the least studied of the three “priority” cations in this set. Li and Zhang (2021) reported $E_{\mathrm{binding}}=-23.56$ kcal mol^−1^ for Cd^2+^@boroxine, with an interaction distance of 2.64 Å [22]. The HOMO–LUMO gap reduction was 61%, smaller than those for Hg^2+^ (65%) and Pb^2+^ (86%). The ordering Cd^2+^ < Hg^2+^ < Pb^2+^ in binding energy, charge transfer, and gap reduction is internally consistent and matches HSAB expectations (Cd^2+^ is a borderline acid; Pb^2+^ and Hg^2+^ are softer). Saad et al. (2022) [29] reported Cd–ligand distances of 3.404–3.428 Å in CdPBCOF-CN complexes, longer than the Pb–ligand distances, again consistent with weaker binding [29].

With only two DFT studies, both on boroxine-based COFs, the Cd^2+^ dataset is too sparse for cross-study comparison. The reported energies are within the chemisorption range, but no study has yet examined pH-dependent speciation (Cd^2+^ hydrolyses above pH ~8) or competing-ion effects at the DFT level.

### 3.4. Chromium (Cr(VI) and Cr(III))

Cr(VI) removal by COFs has been studied both as adsorption and as photocatalytic reduction. Ding et al. (2024) [27] synthesized a cationic porphyrin COF (Imi-TPP-COF-Br-AR) and used M06-2X-D3/def2-SVP for optimization and M06-2X-D3/def2-TZVPD for single-point energies [27]. Two binding modes for Cr_2_O_7_^2−^ were identified; the $E_{\mathrm{interaction}}$ for host–Cr_2_O_7_^2−^-I complex was ~20 kcal mol^−1^ more stable than mode II, attributed to N–H···O and C–H···O hydrogen bonding plus van der Waals interactions. RDG analysis visualized these interactions, and ESP mapping identified the imidazole ring as the primary electrostatic binding site. Experimentally, $q_{\max}=373.14$ mg g^−1^ and LOD = 41 nM. The use of DFT-D3 dispersion correction is appropriate for this system, where non-covalent interactions dominate.

Liu et al. (2021) investigated five COFs with different heterocyclic nitrogen positions for photocatalytic Cr(VI) reduction [37]. Using VASP with PBE-GGA, PAW pseudopotentials, and GGA+U correction ($U_{\mathrm{eff}}=2.5$ eV), they calculated H_2_Cr_2_O_7_ $E_{\mathrm{abs}}$ on heterocyclic N or benzene rings: COF-PDZ (−26.65 kJ mol^−1^) > COF-PMD (−17.37) > COF-PD (−17.18) > COF-1 (−10.55) > COF-PZ (−2.53). The GGA+U correction is important for Cr 3d localization; without it, the adsorption energies would be unreliable. Experimentally, COF-PDZ reduced >95% of Cr(VI) within 20 min (rate constant 0.141 min^−1^), over 23 times faster than COF-1. PDOS analysis showed that heterocyclic N shifted the N 2p states toward the valence band maximum, facilitating visible-light excitation. 

Warrier et al. (2025) synthesized a monolithic triphenylamine COF (BPTF Foam) for oxoanion removal, including CrO_4_^2−^ [38]. B3LYP/def2-TZVP with DFT-D3 gave an $E_{\mathrm{binding}}$ of −17.76 eV for CrO_4_^2−^ versus −66.65 eV for MnO_4_^−^. RDG analysis revealed repulsive (red) regions in the CrO_4_^2−^ interaction that reduce binding strength. Experimentally, $q_{\max}$ was 448.09 mg g^−1^ for CrO_4_^2−^ and 961.54 mg g^−1^ for MnO_4_^−^, with removal within 1 and 3 min, respectively. The very large binding energies (tens of eV) reflect the highly charged nature of the oxoanions and the cluster/periodic model used; they should not be compared directly with the smaller values reported for single-ion adsorption.

The Cr(VI) literature splits into two non-comparable groups: (i) molecular cluster studies of Cr_2_O_7_^2−^ or H_2_Cr_2_O_7_ on finite COF fragments, with dispersion-corrected meta-hybrids; and (ii) periodic studies of CrO_4_^2−^ or H_2_Cr_2_O_7_ on extended COF slabs, often with GGA+U. The reported energies differ by two orders of magnitude, and the underlying physical processes, adsorption versus photocatalytic reduction, are conflated in some papers.

### 3.5. Arsenic (As(III))

Somjit et al. (2022) studied hydroxylated UiO-66 (an MOF, included here because the DFT methodology and As(III) chemistry are directly relevant to COF analogues) [28]. M06-L with LANL2DZ for Zr and As and 6-31G(d,p) for other atoms identified three binding modes. Model 3, with strong covalent As–O bonds (1.71–1.72 Å) to the hydroxylated Zr node, exhibited the best EXAFS fit and a Mulliken charge of 1.29 for arsenic. Experimentally, $q_{\max}=204$ mg g^−1^ over pH 1–10, far exceeding pristine UiO-66 (10 mg g^−1^ for As(III)). The M06-L functional is parameterized for transition metals and is a reasonable choice for Zr; however, the study does not report dispersion corrections, which may affect the physisorption modes (Models 1 and 2).

As(III) is neutral (H_3_AsO_3_) over most of the pH range, so electrostatic adsorption is ineffective and covalent or coordination binding is required. The hydroxylated-node strategy is mechanistically sound, but no equivalent study exists yet on a COF with grafted hydroxyl or thiol sites.

### 3.6. Gold (Au(III))

Gold recovery has attracted substantial DFT interest because Au–S binding is strong, selective, and experimentally well characterized. Abubakar et al. (2025) synthesized a thioanisole-rich COF (TTASDFP) and used VASP with PBE-GGA and PAW pseudopotentials [32]. The material removed >99% of a 9 ppm Au^3+^ solution within 30 s, $q_{\max}=245$ mg g^−1^ and captured gold at concentrations as low as 1 ppb. XPS confirmed Au–S chemisorption. The PBE functional without dispersion correction is adequate for the strong Au–S bond but would be insufficient for the physisorption of AuCl_4_^−^ before reduction.

**Figure 3:**
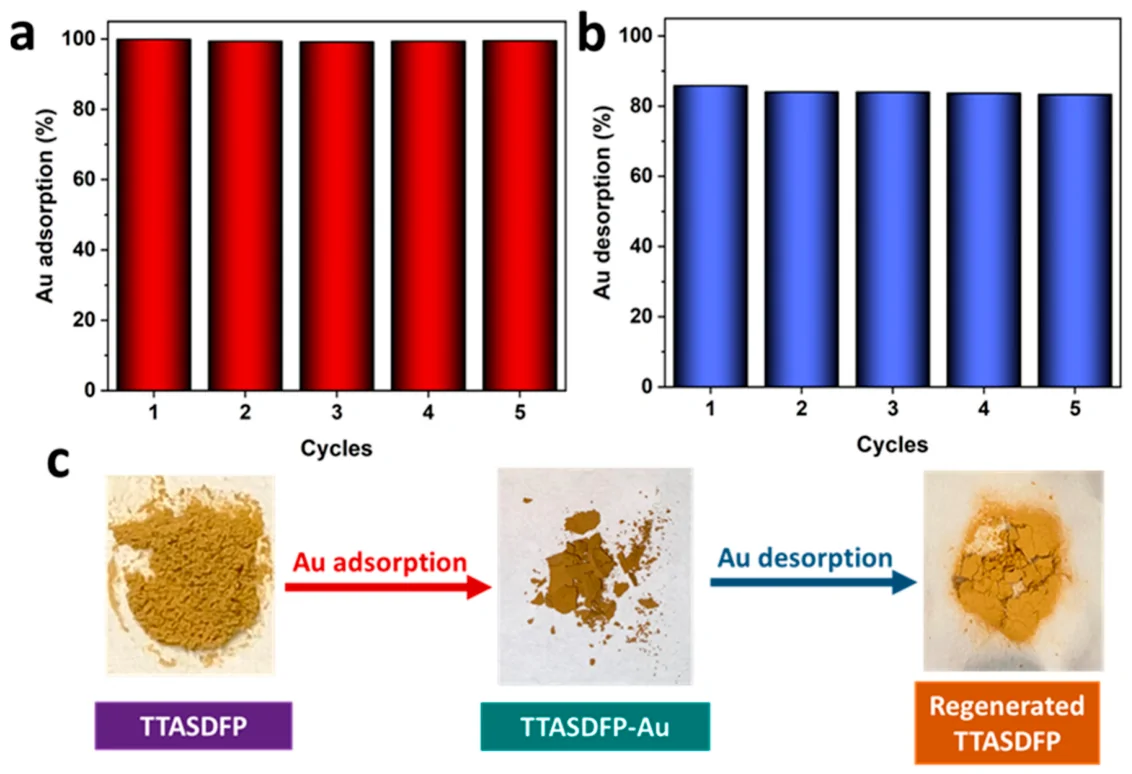
(**a**) Adsorption and (**b**) desorption cycles of gold via TTASDFP. (**c**) Images of TTASDFP powder during the adsorption and desorption cycles (CC-BY license) [33].

Zhong et al. (2022) prepared a hydroxyl-rich COF (TzDa-COF) (Figure 3) and used VASP with PBE-GGA [33]. DFT showed that Au(III) coordinates exclusively through oxygen. Experimentally, $q_{\max}=1866$ mg g^−1^, with 99% removal within 30 min. The mechanism combines coordination and reduction (phenol → benzoquinone), confirmed by PXRD, IR, and XPS. The DFT binding energy is modest compared with the experimental capacity, illustrating that capacity is governed by site density and mass transport, not by the binding energy of a single site.

Wang et al. (2019) modified UiO-66 with thioctic acid (UiO-66-TA) and used B3LYP/LANL2DZ for Au and 6-31+G(d,p) for other atoms [34]. Experimentally, $q_{\max}=374$ mg g^−1^ at pH 2, with high selectivity. The B3LYP/LANL2DZ combination is a common but relatively inexpensive choice; the absence of dispersion correction is less problematic for the covalent Au–S bond than it would be for physisorption.

Zhang et al. (2025) compared COF-NO_2_, COF-NH_2_, and Tu@COF-NH_2_ for Au(III) [36]. ESP analysis showed that electron-withdrawing –NO_2_ reduces local electron density and impedes Au(III) coordination, while electron-donating amino and thiourea groups increase electron density and strengthen binding. The $q_{\max}$ values were 676.2, 1271.3, and 2002.7 mg g^−1^ for COF-NO_2_, COF-NH_2_, and Tu@COF-NH_2_, respectively. The DFT analysis is qualitative, but the trend is clear and consistent with HSAB theory. 

The more important methodological issue is the treatment of AuCl_4_^−^ speciation and the reduction step. Most studies model Au^3+^ or AuCl_3_ rather than the actual solution species AuCl_4_^−^, and few compute the free-energy barrier for the reduction step. The reported binding energies are within the chemisorption range, but the experimental capacities span 245–2003 mg g^−1^, a sevenfold range that reflects differences in site density, pore accessibility, and mass transport, not binding energy alone.

### 3.7. Other Metal Ions (Cd^2+^, Ni^2+^, Cu^2+^, Co^2+^, and Multi-Ion Systems)

Zhu et al. (2023) synthesized a fully π-conjugated COF (Bpy-sp^2^c-COF) with dual binding sites (bipyridine and cyano) and used DMol3 with GGA-PBE [35]. For Hg^2+^, monolayer $E_{\mathrm{ads}}$ values were −2.27 eV (monodentate, N site) and −2.90 eV (bidentate, N=N site); double-layer configurations gave −3.58 and −5.53 eV, respectively. The double-layer energies are more negative, confirming that interlayer adsorption is favored. Experimental $q_{\max}$ values followed the order Hg^2+^ (718.48) > Ni^2+^ (278.64) > Cu^2+^ (260.11) > Co^2+^ (126.23 mg g^−1^), consistent with the DFT trend and with the electronegativity order Hg^2+^ (2.00) > Ni^2+^ (1.92) > Cu^2+^ (1.90) > Co^2+^ (1.88). This study is one of the few that explicitly compares monolayer and interlayer adsorption and validates the DFT ranking against a consistent experimental dataset. 

Zhang et al. [31] used first-principles DFT to study a TOB-DAQ COF coated on graphene oxide (COF-GO-50) for capacitive deionisation of seven heavy metal ions [31]. Two mechanisms were identified: central pore adsorption and carbonyl oxygen complexation. The experimental removal efficiencies exceeded 93% for all ions, with 98% for Cu^2+^ and Fe^3+^. The very small central-pore energies illustrate a common issue: when the adsorption energy approaches zero, the DFT result is dominated by numerical noise and basis-set superposition error, and no reliable mechanistic conclusion can be drawn without counterpoise correction.

Guo et al. (2023) studied an aminal-linked COF membrane (sTPA-PZ) for monovalent/divalent salt separation [30]. DMol3 with GGA-PBE gave an $E_{\mathrm{binding}}$ of 9.2 eV for hydrated SO_4_^2−^ (positive, indicating an energy barrier to entry) versus −0.99 eV for hydrated Cl^−^. The transport energy barriers were 455.5 kJ mol^−1^ for SO_4_^2−^ and 213.2 kJ mol^−1^ for Cl^−^, and MD simulations gave diffusion coefficients of 0.11 and 0.20 Å^2^ ps^−1^, respectively. The membrane achieved 99.1% Na_2_SO_4_ rejection and a Cl^−^/SO_4_^2−^ separation factor of 66. This study is a good example of combining DFT binding energies with MD diffusion coefficients to explain selectivity; the positive binding energy for SO_4_^2−^ is a useful reminder that “adsorption energy” can be positive when the process is barrier-limited rather than spontaneous.

Next, Table 1 summarizes the key DFT parameters and findings for heavy metal adsorption on various COF-based adsorbents. 

## 4. DFT Studies on Radionuclide Adsorption

### 4.1. Uranium (U(VI)) Adsorption on Functionalized COFs

The removal of uranyl ions from aqueous environments has attracted substantial DFT attention, driven by the need for efficient extraction of uranium from nuclear wastewater and seawater. However, the quantitative $E_{\mathrm{ads}}$ vary widely, and this spread is as much a reflection of methodological choices, including the functional, basis set, solvation treatment, and model type. This section therefore treats each study not merely as a source of a numerical value but as a data point for which its interpretation must be conditioned on its computational protocol [39,40,41,42,43,44,45,46,47,48,49,50,51,52,53,54,55,56]. 

Liu et al. (2025) synthesized Tp-PDAN-AO via a two-step method and investigated UO_2_^2+^ coordination using PBE0-D3/6-31G(d)/SDD with the SMD implicit solvation model in Gaussian 16 [39]. Six distinct interaction modes were identified, categorized as η^2^, O-bonding, and chelation modes. The most favorable mode was η^2^ coordination between UO_2_^2+^ and the N and O atoms of the amidoxime group, with a ΔG of −0.585 kcal/mol. The experimental $q_{\max}\;$ was 565.30 mg/g in the dark and 757.62 mg/g under UV irradiation, demonstrating a synergistic photocatalytic reduction pathway. Notably, this study reports a free energy rather than a bare electronic adsorption energy, which makes it one of the few data points in the literature that is directly comparable to experimental spontaneity.

Liu et al. [40] introduced carboxyl groups in situ during LZU-COF synthesis and reported $E_{\mathrm{ads}}$ values of −2.21 eV for UO_2_^2+^ on LZU-COF-COOH versus −1.58 eV on pristine LZU-COF (PBE0-D3/6-31G(d,p)/SDD, Gaussian 16 software) [40]. The ESP maps showed that carboxyl modification introduced a large negative potential region, and PDOS analysis revealed O 2p–U 5f/6d hybridization. The experimental $q_{\max}$ was 136.7 mg/g with equilibrium within 1 h. The ~0.6 eV difference between pristine and functionalized frameworks is consistent with the general trend that added hard-donor groups strengthen binding, but the absolute value should be treated cautiously: this calculation appears to have been performed without explicit solvation, and the reported energy is an electronic E_ads_, not ΔG. 

Zhou et al. (2024) compared amino- and carboxyl-functionalized TzDva COFs using B3LYP/6-31G(d,p) [41]. The carboxyl-functionalized material gave an E_ads_ of −5.72 eV versus −0.14 eV for the amino-functionalized analogue, a striking difference that the authors attributed to the stronger affinity of carboxyl groups for U(VI). The experimental $q_{\max}$ values were 139.5 and 88.3 mg/g, respectively. The very large $E_{\mathrm{ads}}$ for the carboxyl system (−5.72 eV) is almost certainly a consequence of the cluster model and the absence of explicit solvation. 

Guo et al. [42] synthesized TpBpy and TpPy and investigated three adsorption sites (N, O, N=O) using PBE-GGA/VASP [42]. For TpBpy, bidentate N-coordination gave $E_{\mathrm{ads}}$ = −3.23 eV in the monolayer configuration and $E_{\mathrm{ads}}$ = −5.08 eV in the interlayer configuration, the latter attributed to tetradentate coordination between layers. For TpPy, the N=O site gave $E_{\mathrm{ads}}=$ −2.61 eV. Charge transfer was 0.84 e for the O[N] site in TpPy. Experimental $q_{\max}$ values were 115.45 mg/g (TpBpy) and 291.79 mg/g (TpPy). 

Mei and Yan (2023) synthesized TpPa-2OH-Phos and TpDBD-Phos and used PBE0/Gaussian 16 to show that phosphorylation lowers HOMO/LUMO levels and increases the Mulliken negative charge on oxygen, favoring UO_2_^2+^ chelation [43]. The experimental $q_{\max}$ values were 410.9 mg/g and 633.3 mg/g (pH 5), respectively; the superior performance of TpDBD-Phos was attributed to the synergistic effect of phosphonic groups and benzoxazole rings. This study is notable for combining HOMO/LUMO analysis with XPS and DFT to build a consistent mechanistic insight.

Wen et al. (2024) identified oxygen sites in DQTP COF using PBE-GGA/VASP [44]. ESP mapping values showed two negative regions: β-ketoenamine carbonyl (−29.1 kcal/mol) and quinone carbonyl (−32.67 kcal/mol). U–O bond lengths were 2.37 and 2.35 Å, with E_ads_ = −1.33 and −1.32 eV, respectively. Charge transfer was 0.17 e and 0.14 e. The experimental $q_{\max}$ value was 517.62 mg/g at pH 6, with pseudo-second-order kinetics. This study is methodologically careful in that it reports both ESP and E_ads_ values for two distinct oxygen sites, allowing internal comparison. 

Liu et al. (2025) designed a β-keto-enamine Tp-Db COF fluorescent probe and used PBE-GGA/VASP to evaluate five adsorption modes [45]. PDOS revealed U 6d–O 2p and U 5f–O 2p hybridization, while no significant N involvement was observed. The detection limit was 99.34 nM with a 10 s response.

Zhao et al. [46] synthesized Tp-DGCl and BT-DGCl for Pd(II) recovery and used PBE-GGA/DMol3 with B3LYP/def2-TZVP/DFT-D3 for ESP [46]. Model 3, combining electrostatic attraction and N/O chelation, gave the lowest E_ads_ (−37.24 kcal/mol, Tp-DG_Cl_). The experimental Pd(II) $q_{\max}$ values were 342.1 (Tp-DG_Cl_) and 231.1 (BT-DG_Cl_) mg/g. While the target metal is Pd rather than U, the study is included here because it demonstrates the same methodological pattern, a single low-energy model being used to argue for a mixed electrostatic/coordination mechanism, and because it provides a useful comparison point for the relative strength of soft-metal versus hard-metal binding.

He et al. (2025) constructed NCOF-3 for photocatalytic U(VI) reduction [47]. ESP maps showed negative regions concentrated around Cl and imine-N, and HOMO/LUMO localization confirmed the D–A character. The photocatalytic removal capacity was 1084 mg/g. This study is mechanistically distinct from adsorption-only studies: the DFT descriptors are photocatalytic, not adsorptive, and the reported capacity reflects a combined adsorption–reduction process. 

Sun et al. (2024) used PBE0/SDD/Gaussian 16 with ETS-NOCV analysis to show that sulfonate groups bind uranyl through electron transfer from U 7s/5f to S/O 2p orbitals [48]. The membrane reached equilibrium in 10 min with $q_{\max}$ of 358.8 mg/g at pH 6. The ETS-NOCV analysis provides a level of electronic-structure detail that is rare in the literature and supports a covalent/coordination rather than purely electrostatic mechanism.

Liu et al. [49] grew a β-ketoenamine COF on carbon nanotubes and used DFT (Multiwfn) to show that UO_2_^2+^ E_ads_ values were −24.22 kJ/mol on COF-OH and −39.17 kJ/mol on CNT/COF-OH [49]. Cyclic voltammetry gave reduction potentials of −0.53 and −0.58 V vs. SHE, below the U(VI)/U(IV) value of 0.411 V, confirming thermodynamic feasibility of reductive immobilization. The experimental $q_{\max}$ value was 518.2 (CNT/COF-OH) mg/g. 

### 4.2. Technetium (TcO_4_^−^) Adsorption on Cationic COFs

Zhao et al. (2022) combined DFT (PBE-GGA/DFT-D3/VASP) and molecular dynamics to study three imine-linked cationic COFs (DBBT, DBTFP, and PITFP) for ^99^TcO_4_^−^ removal [50]. The ESP maximum value on the positively charged N of the MTHP group was 95.69 kcal/mol, which was identified as the preferred adsorption site. DBBT-AA outperformed DBBT-AB and the other frameworks; the carbonyl group in DBTFP had a slightly negative effect, while the small pore size of PITFP was detrimental despite the presence of more adsorption sites. Increasing the TcO_4_^−^ concentration accelerated adsorption but did not achieve full elimination due to the difficult desorption of inner-layer counter-anions. This study is unusual in the reviewed set in that it uses MD to capture dynamic anion-exchange behavior, partially addressing the static-model limitation discussed in Section 2.3.

### 4.3. Palladium (Pd(II)) Adsorption for Radionuclide Separation

Wang et al. (2024) synthesized COF-SSH with thiol and thioether groups and used PBE-GGA/DFT-D3/VASP to calculate E_ads_ values of Pd(NO_3_)_2_ at the S-H (−2.63 eV), S (−2.20 eV), and N (−1.65 eV) sites [51]. The S-H site was identified as the dominant adsorption site, and the experimental $q_{\max}$ was 420.2 mg/g, with equilibrium reached within 10 min.

**Figure 4:**
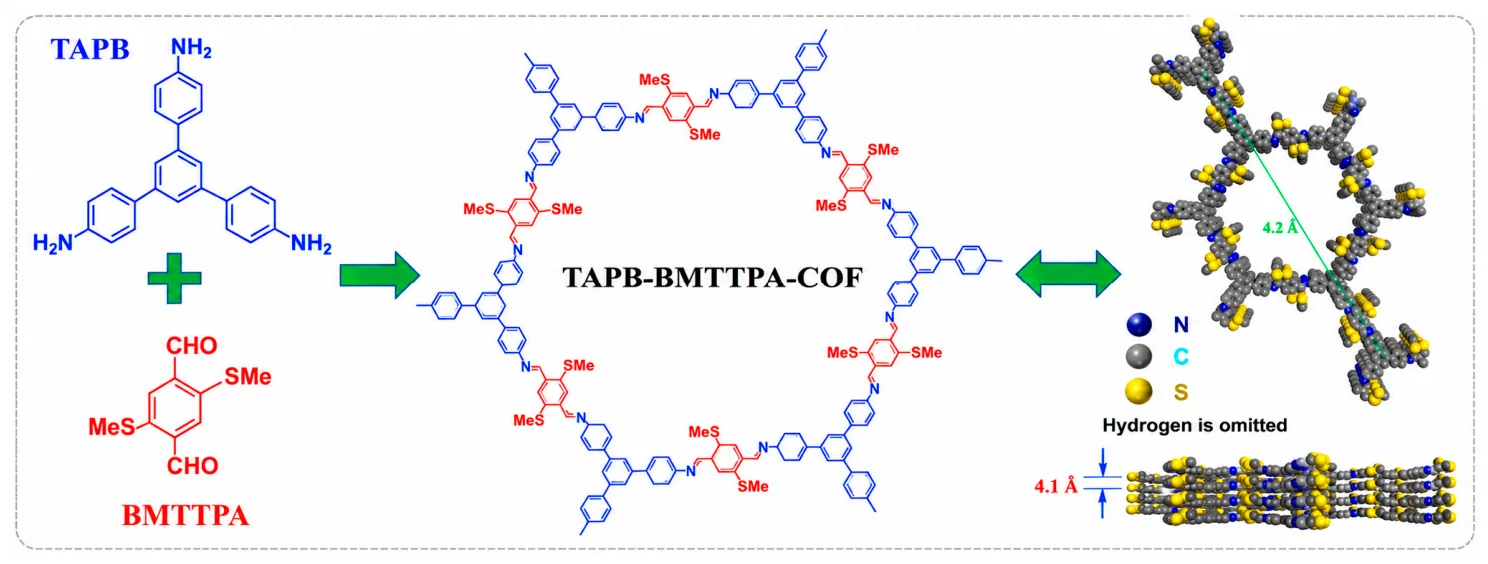
Synthetic scheme of TAPB-BMTTPA-COF and its graphic view (CC-BY license) [52].

Wang et al. (2025) reported a similar sulfur-rich TAPB-BMTTPA-COF (Figure 4), with Pd and E_ads_ values of −2.00 eV (S) and −1.83 eV (N) and $q_{\max}$ values of 343.6 mg/g [52]. The consistency between these two studies was noted, and both studies used PBE-GGA/DFT-D3/VASP. 

**Figure 5:**
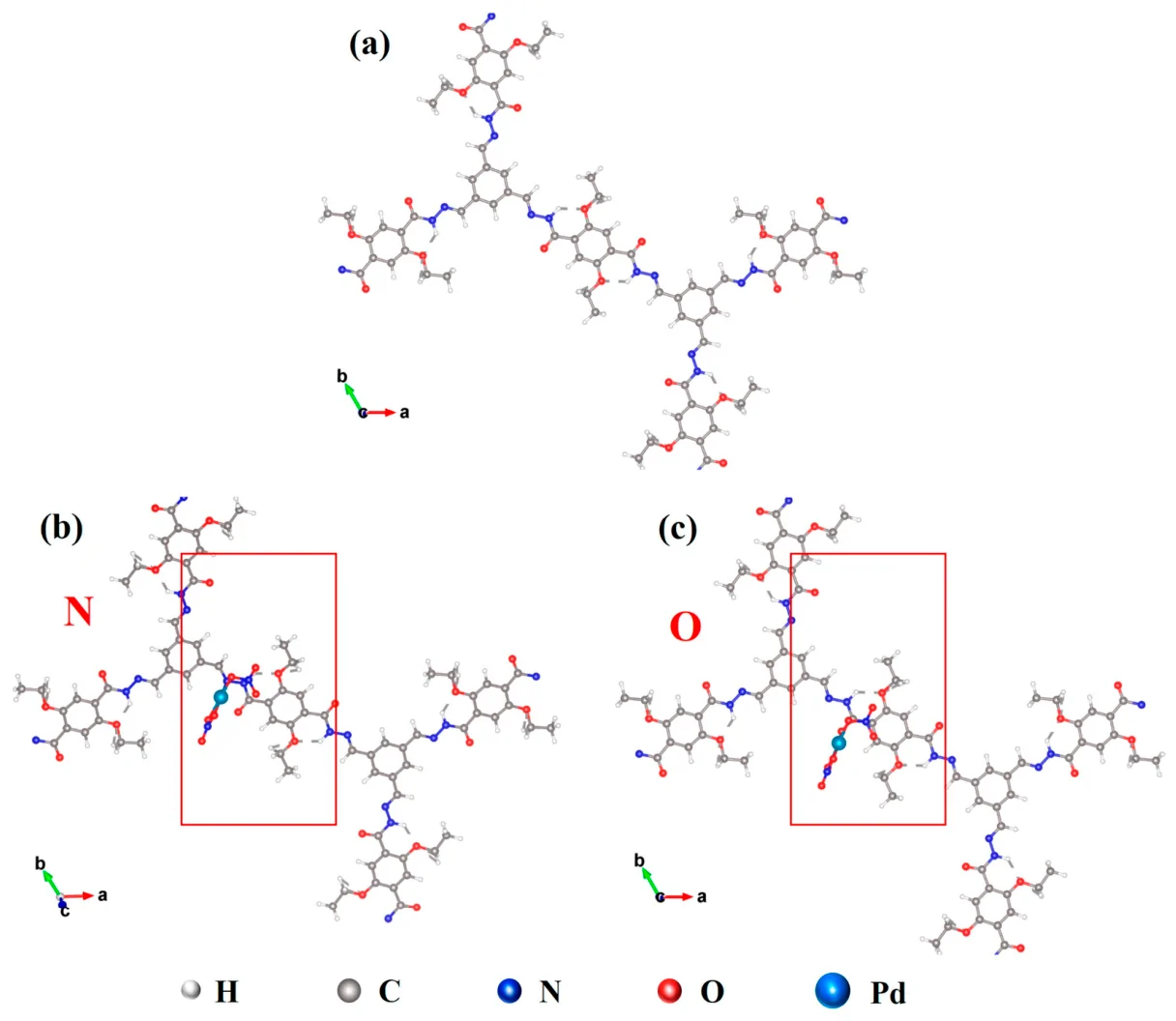
(**a**) DFT calculations of COF-42 and Pd(NO_3_)_2_ with (**b**) N and (**c**) OonPd@COF-42 structure. The red box stands for the binding site (CC-BY license) [53].

Song et al. [53] used PBE-GGA/DFT-D3/VASP to study COF-42 (Figure 5) for Pd adsorption from high-level liquid waste, reporting E_ads_ values of −1.75 eV (N) and −1.67 eV (O), with $q_{\max}$ values of 170.6 mg/g at 3 M HNO_3_ [53].

Junli et al. [54] reported DHTP-TPB COF with Pd E_ads_ values of −1.89 eV (N), −1.17 eV (NH), and −0.94 eV (OH) and $q_{\max}$ values of 142.8 mg/g [54]. The N-site preference is consistent across both studies, but the absolute values differ by ~0.14 eV, a difference that is within the expected functional/pseudopotential uncertainty discussed in Section 2.3.

Hu et al. (2025) used DFT to study Pd nanoclusters within TpPz COF membranes, reporting an E_ads_ of −0.70 eV for active H and ELF evidence of strong Pd–pyrazine N affinity [55]. This study is included because it demonstrates the use of DFT to rationalize catalytic rather than purely adsorptive behavior and because the Pd loading (4.0%) and nanocluster size (1.96 nm) provide a bridge between atomistic modeling and membrane-scale performance.

### 4.4. Radioactive Iodine (^131^I) Adsorption on Vinyl-Functionalized COFs

Tian et al. (2023) synthesized vinyl-functionalized spherical COF-V and used M06-2X/def2-SVP/Gaussian 16 with IGMH and AIM analysis to study I^−^ adsorption [56]. E_binding_ values were −10.4 kcal/mol (COF-V with vinyl), −9.1 kcal/mol (TAPT-PDA with triazine), and −8.5 kcal/mol (TAPB-PDA without vinyl). PDF analysis showed I^−^–benzene interactions at 1.58 Å and I^−^–vinyl interactions at 3.12 Å, supporting a hydrogen-bonding mechanism (C–H···I^−^) rather than purely electrostatic capture. The experimental $q_{\max}$ value was 437.8 mg/g, with >90% removal of Na^131^I at activities up to 1017.5 MBq. This study is methodologically distinctive in its use of PDF and IGMH to resolve the interaction geometry, and it provides one of the few examples in this review where the computed binding energy is small enough to be physically plausible as a physisorption/hydrogen-bonding interaction without requiring large solvation corrections.

Table 2 summarizes the key DFT parameters and findings for radionuclide adsorption on various COF-based adsorbents. 

## 5. DFT Studies on Organic Pollutant Removal

This section reviews DFT studies of organic pollutant removal by COFs, covering antibiotics, pharmaceuticals, per- and polyfluoroalkyl substances (PFAS), dyes, flame retardants, pesticides, and other emerging contaminants [57,58,59,60,61,62,63,64,65,66,67,68,69,70,71,72,73,74,75,76,77,78,79,80,81,82,83,84,85,86,87,88]. 

A recurring problem in this literature, and one that shapes the discussion below, is that adsorption energies are routinely presented as if they were directly comparable across studies and directly translate into adsorption capacity. The reviewed studies employ GGA, meta-GGA, hybrid, and tight-binding functionals; DFT-D2, DFT-D3, DFT-D3(BJ), DFT-D4, and, in some cases, no dispersion correction; implicit solvation, explicit solvation, and vacuum; periodic slabs and finite clusters; and both electronic E_ads_ and ΔG_ads_. For physisorbed organic pollutants, the choice of the dispersion scheme alone can shift the computed binding energy by 20–100 kJ/mol, which is often greater than the binding energy itself. The numerical values reported below should therefore be read as mechanistic indicators within each study, not as a cross-study ranking of COF performance. Where a study reports ΔG_ads_ with explicit thermal and solvation corrections, this is noted because such values are the only ones that can be meaningfully connected to aqueous-phase spontaneity.

### 5.1. Antibiotics Removal

Zhuang et al. [57] examined SMT adsorption on TPB-DMTP-COF using DFTB+ with the 3ob parameter set for the framework and DFT-COSMO (DMol3, GGA-PBE/DNP with Grimme dispersion) for charge distributions. Herein, SMT is trapped in the pore rather than on the top surface, with the benzene ring parallel to the pore wall at a vertical separation of 0.27 nm and C–H···π contact as the dominant interaction is a qualitative geometric result and is therefore relatively robust to functional choice. The reported adsorption energies, however, were not accompanied by explicit solvation or thermal corrections, so they describe 0 K electronic binding rather than aqueous-phase spontaneity. The experimental $q_{\max}$ value of 209 mg/g at natural pH is consistent with pore-site capture, but the study does not establish a quantitative link between the computed energetics and the measured capacity. 

The most instructive study in this group is that of Liu et al. [83], who deliberately varied nanopore size across three β-ketoenamine COFs (TpPa-1, TpBD, and TpTph) and used periodic DFT (VASP and PAW-PBE) to identify site-specific binding energies and adsorption probabilities. The result that TpBD, with an intermediate pore size, outperforms both the smaller-pore TpPa-1 and the larger-pore TpTph is mechanistically significant: It shows that adsorption capacity is governed not by the single strongest binding site but by the number of accessible sites per unit pore volume combined with the binding energy distribution. The reported site $E_{\mathrm{ads}}$ values span from −0.253 to −54.31 kJ/mol across the three frameworks, with the highest-probability site on TpTph (−54.31 kJ/mol, 99.99% probability) being the strongest single interaction but not producing the highest capacity. This decoupling between maximum site binding energy and macroscopic capacity is one of the clearest demonstrations in the reviewed literature that a single E_ads_ value cannot predict adsorption performance. The calculations were performed without explicit solvation, and the adsorption probabilities are statistical weights over static configurations rather than thermodynamic populations, so the numerical values should be treated as relative indicators within this series.

Huang et al. [60] investigated an NH_2_-MIL-125@SNW-1 MOF@COF composite for tetracycline degradation and used periodic DFT (DMol3) to establish the charge-transfer direction at the interface. The DOS analysis, assigning the valence band of the MOF to O 2p, the conduction band edge to Ti 3p, and the corresponding assignment for the COF to C 2p and N 2p, is a robust qualitative result that supports the proposed Z-scheme mechanism. The 3D charge density difference confirming electron transfer from MOF to COF is similarly qualitative. The reported 93.1% removal under UV light is therefore mechanistically suggestive but not quantitatively predicted by the DFT results.

Liu et al. [58] studied COF-PRD (pyridine-based COF) for the degradation of bisphenol A (BPA) and other bisphenols under visible light with peroxymonosulfate (PMS) activation. Herein, DFT calculations were performed using Gaussian 16 at the B3LYP/6-31G(d) level, and Fukui indices were used to identify reactive sites for nucleophilic, electrophilic, and radical attacks on bisphenol molecules. The calculations revealed that C3 and C4 atoms with high f^0^ values (0.064 and 0.062 for BPA) were susceptible to superoxide radical attack, initiating hydroxylation reactions. The study demonstrated that the degradation kinetics followed the order BPB ≈ BPF ≈ BPA > BPZ > BPAP, which was rationalized through DFT-based analysis of steric hindrance effects and electron-donating capabilities of substituent groups. The degradation pathway analysis revealed that the main degradation pathway included hydroxylation, electrophilic attack, and ring-opening reactions.

Lin et al. [61] combined experiments with cluster DFT (Gaussian 09, B3LYP-D3/6-31G(d)) for four fluoroquinolones on NH_2_-MIL-125@TpPa-SO_3_H. This study is methodologically stronger than most in the group because it reports NBO charge transfer (0.153 e from enoxacin to the COF), explicit interaction distances (1.627, 2.244, and 1.787 Å for the three hydrogen bonds), and an energy decomposition analysis attributing the binding to electrostatics with a secondary dispersion contribution. The $q_{\max}$ values of 444.7–457.5 mg/g across the four FQs are comparable to the DFT-predicted ordering. Two caveats apply. First, the cluster model truncates the COF, removing interlayer interactions and long-range electrostatics; the reported binding energies may therefore differ from periodic values for the same system. Second, the calculations were performed in implicit solvent without explicit pH-dependent speciation of the zwitterionic FQs, whereas the experimental optimum at pH 6 reflects a specific speciation distribution. 

### 5.2. Pharmaceutical Pollutants Removal

Jin et al. [59] reported one of the few studies in this review that combines an explicit free-energy treatment with a well-defined pH. For naproxen on guanidinium-based TFPB-TgCl, the M06-2X/def2-SVP + D3(BJ) calculations in Gaussian 09 yielded an $E_{\mathrm{binding}}$ of −131.57 kcal/mol for the electrostatic guanidinium–carboxylate configuration, with IGMH analysis identifying van der Waals contacts as the secondary interaction and ESP mapping confirming the electrostatic complementarity. The experimental $q_{\max}$ value of 308 mg/g at pH 5 is consistent with the proposed mechanism. The very large binding energy, however, reflects the gas-phase or implicitly solvated ion-pair interaction and should not be interpreted as an aqueous-phase adsorption free energy; in water, the desolvation penalty for the carboxylate and guanidinium groups would substantially reduce the net driving force. 

Ghasemi et al. [62] used MD simulations with DFT validation (Gaussian 09, B3LYP/6-31+G) to compare three COFs against six pharmaceuticals. The DFT component serves here as a cross-check on the force-field-derived interaction energies rather than as an independent prediction, and the reported $E_{\mathrm{ads}}$ values (−0.81 to −1.18 eV) are consistent with the MD ranking. The mechanistic conclusion—that van der Waals interactions dominate over electrostatics for these neutral and weakly charged pharmaceuticals—is supported by the force-field decomposition and reproduced by the DFT single-point energies. The study does not, however, report ΔG_ads_, and the comparison across COFs is limited by the use of a single functional and basis set without a systematic convergence check.

Akhzari et al. [63] extended this approach to macrolides and dexamethasone on TAPB-DMTP COFs using well-tempered metadynamics in addition to MD. The Δ*G* landscapes, with global minima of −665.81 kJ/mol for dexamethasone, −638.53 kJ/mol for azithromycin, −566.31 kJ/mol for erythromycin, and −326.75 kJ/mol for clarithromycin on the hydroxyl-functionalized COF, are among the few genuinely free-energy quantities reported in this literature and are therefore more directly comparable to experiment. The mechanistic attribution to hydrogen bonding and X–H···π interactions is supported by hydrogen-bond counting and RDF analysis. The caveat is that the metadynamics free energies are computed with a classical force field rather than with DFT, so they inherit the force-field parameterization; the DFT contribution in this study is limited to the electronic structure of the isolated components rather than the adsorption complex.

Khedri et al. [86] employed MD simulations and DFT calculations to assess the adsorption of phenazopyridine (PHP), an analgesic drug, on three MOFs (IRMOF-16, HKUST-1, and ZIF-8) and COFs. Their DFT calculations using Gaussian 09 at the B3LYP/6-31+G* level revealed that the adsorption energies for IRMOF-16, HKUST-1, and ZIF-8 were −1.53, −1.31, and −0.04 eV, respectively, confirming the superior PHP adsorption capacity of IRMOF-16. The study demonstrated that PHP adsorption intensity followed the order IRMOF-16 (100%) > HKUST-1 (60%) > ZIF-8 (40%). The DFT results also showed that functionalization of IRMOF-16 with hydroxyl groups and hybridization with COF improved the adsorption capability due to negative net charge enhancement on the surface.

Sheng et al. [85] synthesized charged COF membranes for organic chloramine precursors removal and employed DFT calculations to investigate the rejection mechanism. Their DFT calculations using Gaussian 16 at the B3LYP-D3BJ level with mixed basis sets (6-31++G(d,p) for oxygen, 6-31G(d,p) for other atoms) revealed that the addition of sulfonate groups significantly increased the negatively charged area of COF channels. The migration energy barriers for serine transferring through TpPa and TpPa-SO_3_H nanochannels were calculated, showing that TpPa-SO_3_H exhibited a much higher peak (3.49 eV) compared to TpPa (0.18 and 0.36 eV), indicating that solid electrical repulsion due to sulfonate groups played a critical role in removal efficiency enhancement. The study demonstrated that the combined application of KMnO_4_ pre-oxidation and PA-TpPa-SO_3_H/PAN membrane nanofiltration could minimize the formation of organic chloramines in subsequent chlorination.

Wang et al. [82] prepared a new covalent organic framework embedded with silver nanoparticles (Ag@TPHH-COF) and employed DFT calculations to investigate its catalytic performance for degradation of organic pollutants. Their DFT calculations, performed using the Materials Studio suite, revealed that the activation energy for the catalytic reduction of 4-nitrophenol was calculated to be 17.44 kJ/mol, significantly lower than that previously reported for Ag nanoparticle catalysts. The study demonstrated that Ag@TPHH-COF could catalyze the reduction of various nitroaromatic compounds and organic dyes with high efficiency. The TOF value for the catalytic reduction of 4-NP was 4.15 min^−1^. The DFT results confirmed that the silver nanoparticles (diameters of 5±3 nm) were uniformly dispersed on the surface and in the interlayer gaps of the TPHH-COF substrate, with the nitrogen and oxygen atoms in the COF contributing largely to anchoring Ag^0^.

### 5.3. Per- and Polyfluoroalkyl Substance (PFAS) Removal

PFAS removal is the area where the distinction between anion exchange and hydrophobic or fluorophilic association is most consequential and where DFT has been most useful in separating the two contributions. 

Wang et al. [64] used cluster DFT (Gaussian 09, B3LYP/6-31G) to decompose the adsorption of GenX and HFPO-TA on a cationic COF into an initial electrostatic attraction step (Δ$E_{\mathrm{binding}}$ ≈ −82 kcal/mol for both) and a subsequent ion-exchange step that is endothermic for GenX and nearly thermoneutral for HFPO-TA. This decomposition is mechanistically valuable because it explains the experimentally observed competitive replacement of GenX by HFPO-TA: the two adsorbates are nearly indistinguishable in the initial electrostatic step, and selectivity is determined entirely by the ion-exchange step.

Wang et al. [65] reported β-cyclodextrin COFs for PFOS, PFHxS, PFBS, and F53B, with ORCA B97-3c calculations yielding a Δ$E_{\mathrm{binding}}$ of −37.93 kcal/mol for PFOS in the β-CD cavity and an IGM analysis identifying N–H···O–S hydrogen bonds between the sulfonate head and the amino groups, together with van der Waals contacts between the C–F chain and the ether rings. The binding-energy ordering (PFOS > F53B > PFHxS > PFBS) matches the experimental adsorption capacities, providing a comparison between the computational ranking and the experimental data. The study is careful to note that the calculated ΔE_binding_ values neglect interlayer interactions along the *z*-axis of the crystal cell, which is an explicit statement of the model limitation. 

Wang et al. [66] addressed the fluorophilicity hypothesis directly by comparing the binding of PFOS and PFHxS to the C–F chain versus the hydrocarbon portion of a fluorinated quaternary ammonium COF and by comparing the binding of the hydrocarbon analogues SHS and SOS to the same two regions. The result that PFASs bind preferentially to the C–F chain (Δ$E_{\mathrm{binding}}$ = −11.0 kJ/mol for PFOS, Δ$E_{\mathrm{binding}}$ = −8.7 kJ/mol for PFHxS), while hydrocarbons bind preferentially to the hydrocarbon portion, provides a computational comparison of fluorophilic selectivity. This is one of the more mechanistically complete PFAS studies in this review. The B3LYP/6-31G level with an IEFPCM solvation model is modest by current standards, and the absence of a dispersion correction beyond what is implicit in the functional is a limitation for a system dominated by van der Waals and fluorophilic interactions.

Zhang et al. [67] extended the PFAS work to a chitosan–COF composite, reporting B3LYP/6-311G(d) Δ$E_{\mathrm{interaction}}$ of 33.2 kJ/mol for COF–PFOA and 23.8 kJ/mol for chitosan–PFOA, rising to 45.3 kJ/mol for protonated chitosan. The mechanistic conclusion—that the quaternary amine on the COF is the primary adsorption site while the protonated amine on chitosan provides a pH-dependent secondary contribution—is supported by ESP mapping and IRI analysis and explains the experimentally observed pH dependence of the composite. The study is also one of the few to combine DFT with MD simulations of the composite architecture, showing that chitosan adheres to the COF exterior rather than penetrating the pores. The interaction energies are small in absolute terms and were computed without explicit solvation; the pH-dependent trend, however, is robust because it follows from the protonation state rather than the absolute energy scale.

### 5.4. Dyes and Organic Pollutants Removal

Kumar et al. [68] used periodic DFT (VASP and PBE-D3) to rank five cationic dyes on an amorphous tetrazine–triazine COF, obtaining binding energies from −1.41 eV for methylene blue to Δ$E_{\mathrm{binding}}$ = −0.79 eV for rhodamine B, in agreement with the experimental removal order. The mechanistic attribution to a combination of electrostatic, π–π, and hydrogen-bonding interactions is consistent with the ESP and IGM analysis. The study is notable for using an amorphous rather than crystalline framework model, which is closer to the experimental material, although amorphization was not parameterized against a measured defect density. 

Li et al. [69] combined GCMC simulations with DFT for malachite green on a cage COF, reporting an isosteric heat of −76.03 kcal/mol for MG versus −70.14 kcal/mol for rhodamine B and −46.23 kcal/mol for methyl violet, and diffusion coefficients showed the slowest diffusion for MG. The isosteric heat is a more experimentally meaningful quantity than a static E_ads_ because it includes the temperature dependence of the adsorption equilibrium, and its ordering matches the experimental selectivity. The DFT contribution here is limited to the framework electronic structure rather than to the adsorption complex, so selectivity is attributed primarily to geometric and electrostatic factors derived from force-field simulations.

Chen et al. [70] used DMol3 calculations to demonstrate charge-controlled separation on an ionic COF membrane, obtaining Δ$E_{\mathrm{interaction}}$ values of −0.72 eV for cationic methylene blue, +12.29 eV for neutral nile red, and +36.82 eV for anionic methyl orange. The sign reversal across the three charge states is the key mechanistic result and is robust to the choice of functional group because it reflects the dominant electrostatic term. The magnitudes, however, should not be compared directly with binding energies from other studies because the membrane model is periodic and the values include the full framework response. The study demonstrates that the anionic sulfonate sites are responsible for charge selectivity, a conclusion that is consistent with the experimental rejection data (>99% for cationic dyes, partial rejection for anionic dyes). 

Wei et al. [71] combined experiments with periodic DFT (VASP and PBE-D3) and MD for BPA on magnetic Fe_3_O_4_@TpND. The DFT result that BPA binds preferentially to the phenolic aldehyde groups with an $E_{\mathrm{ads}}$ of −7.65 kcal/mol, together with the charge density difference showing electron transfer from the BPA hydrogen atoms to the carbonyl oxygen, provides a hydrogen-bonding mechanism. The MD simulations add the observation that BPA aggregates at high concentrations and that the pore structure, rather than the surface sites alone, governs uptake. The study distinguishes the electrostatic contribution, which is not dominant, from the hydrogen-bonding contribution, which is, and the XPS shift of the carbonyl peak supports the proposed binding mode. The adsorption energy is small, consistent with physisorption, and the experimental $q_{\max}$ of 114.97 mg/g at 298 K is moderate. The absence of explicit solvation means the computed energy corresponds to a dehydrated binding site, which, for a hydrogen-bonded complex, can differ substantially from the aqueous value.

Liu et al. [72] examined side-chain functionalization of COFs with electron-withdrawing groups for photocatalytic BPA degradation using ORCA B3LYP/def2-SVP calculations. The key result—that CN-COF exhibits the broadest ESP value range (−36.94 to 27.63 kcal/mol) and the highest photocatalytic rate constant (0.94 min^−1^)—is a structure–activity correlation rather than an adsorption energetics result, and it is supported by photoelectrochemical measurements. The mechanistic interpretation—that the moderate electron-withdrawing strength of the nitrile group optimizes the balance between charge separation and recombination—is consistent with PL, EIS, and transient absorption data. The DFT contribution here is the ESP and frontier-orbital analysis, which is a qualitative descriptor.

Hao et al. [73] used ONIOM QM/MM calculations (B3LYP-D3/6-31G(d):UFF) for BPA, BPB, BPF, diclofenac, and sulfamethazine on PyTTA-Dva-COF, obtaining $E_{\mathrm{ads}}$ values of 53.35, 55.86, 55.56, 23.51, and 14.89 kJ/mol, respectively. The ordering, with the bisphenols binding more strongly than diclofenac and sulfamethazine, is attributed to the formation of two hydrogen bonds per bisphenol molecule versus one for the other pollutants. The RDG analysis supports the assignment of hydrogen bonding and van der Waals interactions as the dominant terms. The ONIOM approach allows a larger model than a pure cluster calculation while retaining hybrid-functional accuracy in the binding region, which is a methodological advantage. 

Shen and Yan [74] studied azo-functionalized COFs for photo-modulated adsorption and sensing of aniline. Their DFT calculations at the B3LYP/6-311G(d,p) level revealed that the minimum value of ESP at the position of the azo bond markedly decreased after 365 nm irradiation, while the corresponding dipole moment increased. The energy gap between HOMO and LUMO of the cis-azo configuration (3.56 eV) exceeded that of the trans-azo form (2.64 eV), explaining the blueshift phenomenon in the emission maximum upon irradiation. The fluorescence quenching mechanism was attributed to photoinduced electron transfer (PET), where the HOMO energy level of aniline lay between the LUMO and HOMO of ThTFB-3N=N, facilitating electron transfer from the HOMO of aniline to the HOMO of ThTFB-3N=N. The study demonstrated that cis-ThTFB-3N=N exhibited a nearly tenfold enhancement in adsorption capacity for aniline (303.3 mg/g) compared to the trans form (30.7 mg/g).

Zhao et al. [75] explored the interaction mechanism of polystyrene nanoplastics with TAPB-TPA COF using DFT and MD simulations. Their DFT calculations, performed using the DMol3 module in Materials Studio at the PBE-GGA/DND level, revealed that the adsorption energy was -1.04 eV, confirming strong adsorption interactions between PSNPs and COF. The electron cloud distribution and electrostatic potential analysis showed that the overall positively charged COF was prone to adsorb negatively charged PSNPs. The HOMO-LUMO analysis showed that COF (ΔE = 1.982 eV) had a lower energy gap than PSNPs (ΔE = 4.189 eV), indicating more favorable electron transfer. MD simulations demonstrated that non-bonding interactions (van der Waals forces, electrostatic interactions) were significantly stronger than chemical bonding interactions, suggesting physisorption as the primary mechanism. The radial distribution function (RDF) analysis showed no significant peaks within 3.5 Å, confirming that non-bonding interactions were dominant.

Mokhtari et al. [84] synthesized a hydrazone-linked COF (Hz-TPFTZ-COF) and incorporated it into PSf/PVP mixed matrix membranes for dye wastewater purification. DFT simulations revealed a staggered stacking structure with micropores of 1.3 and 2.1 nm, consistent with PXRD and BET results (145 m^2^/g). Experimentally, adding 1.0 wt% COF increased tensile strength from 2.26 to 3.43 MPa, reduced pure water permeability from 373.84 to 94.69 L·m^−2^·h^−1^·bar^−1^, and achieved dye rejections of 92% (BB41) and 88% (MV) at pH 7.

### 5.5. Flame Retardants and Other Emerging Contaminants

Wang et al. [76] reported that pore size, rather than maximum binding energy, controls capacity. For triphenyl phosphate (TPhP) on three COFs with increasing pore size, the VASP PBE sorption energies were 0.045, 0.423, and 0.172 eV for COF1 (smallest pore), COF2 (intermediate), and COF3 (largest), respectively, matching the experimental capacity order COF2 > COF3 > COF1. The mechanistic explanation—that TPhP cannot enter the smallest pore and escapes too easily from the largest—is supported by the optimized adsorption geometries. The study illustrates a case where the ranking of computed energies across a series of related materials is mechanistically informative, even though the absolute values are not directly comparable to solution-phase adsorption free energies.

He et al. [77] investigated phytic acid on Fe-, Ni-, Fe–Ni-, and Zn-incorporated COFs using DMol3 GGA-BLYP calculations, obtaining $E_{\mathrm{ads}}$ values of −1.42, −0.99, −0.84, and −0.78 eV, respectively, with the ordering Fe > Ni > Fe–Ni > Zn > pristine COF. The mechanistic attribution to multidentate coordination combined with electrostatic attraction is supported by the ESP analysis and by the frontier-orbital result showing that the Fe/COF HOMO lies above the phytic acid LUMO, enabling electron transfer. The study indicates that the metal d-electron configuration affects the binding strength, with the fully occupied Zn 3d^10^ configuration unable to participate in chelation. The experimental $q_{\max}$ of 615.38 mg/g for Fe/COF is among the higher values in this review for an organophosphorus pollutant. The calculations are periodic and include spin polarization, which is appropriate for the magnetic Fe centers, but they do not include explicit solvation or thermal corrections. In addition, comparison across the metals is affected by differences in the ionic radii and hydrolysis behavior of the four cations in water.

Cheng et al. [78] used DFT to study aryl organophosphate esters on a heteropore COF, reporting HCOF $E_{\mathrm{ads}}$ from −2.8 to −16.7 eV and melamine sponge (MS) $E_{\mathrm{ads}}$ from −0.6 to −5.5 eV. The large spread reflects differences in the molecular sizes and functional groups of the nine target analytes, and the comparison between HCOF and MS is used to argue that the heteropore architecture provides stronger binding. The study provides less mechanistic detail than others in this group, and the reported energies are not accompanied by a decomposition into electrostatic, dispersion, and hydrogen-bonding contributions. The XPS evidence for π–π and hydrogen-bonding interactions is consistent with the DFT results, but the very large binding energies (up to −16.7 eV) are difficult to reconcile with the experimental adsorption behavior and may reflect the absence of solvation and the use of a cluster model that does not account for full framework relaxation.

Liu et al. [79] reported a mechanistic study of polybrominated diphenyl ethers (PBDEs) on a triazine-cored COF. The DMol3 GGA-PBE $E_{\mathrm{ads}}$ values for BDE28, BDE47, BDE99, BDE100, and BDE154 (−27.01, −31.06, −35.92, −36.20, and −48.47 kJ/mol, respectively) correlate linearly with the number of bromine atoms (R^2^ = 0.8927), and the IGM analysis identifies circular blue isosurfaces between the N atoms of the triazine or imine groups, the O atoms of the methoxy groups, and the Br atoms of the PBDEs. This provides a computational assignment of halogen bonding as the dominant interaction and shows a linear correlation between binding energy and bromine content. The study illustrates how DFT can be used to identify a specific interaction type and examine its dependence on molecular structure.

### 5.6. BTEX and Hydrocarbon Removal

Li et al. [80] used periodic DFT (VASP and GGA-PBE) to study BTEX on a thiol-functionalized COF, obtaining Langmuir-type adsorption energies of −150.2 to −255.8 kJ/mol, with the ordering o-xylene > ethylbenzene > p-xylene > m-xylene > toluene > benzene. The mechanistic attribution to π–π stacking and hydrophobic interactions between the conjugated aromatic framework and the BTEX molecules is consistent with the experimental Langmuir fits (R^2^ > 0.99) and with the simultaneous uptake of Hg(II) by the thiol groups. The study shows that the BTEX and Hg(II) binding sites do not interfere, which is relevant to the design of multifunctional adsorbents. The reported energies are large for what is described as physisorption, which may reflect both the periodic model and the absence of solvation; in water, the hydrophobic driving force would be partially offset by the desolvation penalty.

Inah et al. [81] used cluster DFT (Gaussian 09, B3LYP/6-311++G(2d,2p)) to study PTFE-decorated and metal-doped COFs for BTEX, reporting $E_{\mathrm{ads}}$ values in the range of 0.00265–0.00274 MeV (approximately 2.65–2.74 eV, although the use of MeV is unusual and should be verified against the original units) and band gaps ranging from 0.146 eV for PTFE-COF to 5.5 eV for the Li-doped ethylbenzene complex. The study includes d-band center analyses for the Fe-doped systems (0.2–0.9 eV) and the Li-doped systems (4.1–4.5 eV), which provide a descriptor for transition-metal chemisorption. The mechanistic conclusion that Fe doping enhances chemisorption while Li doping has a mixed effect is supported by NBO and QTAIM analyses. The use of a cluster model for a polymer-decorated framework is a substantial simplification, and the reported adsorption energies are not directly comparable to those from periodic slab calculations.

### 5.7. Photocatalytic Degradation Studies

Zhao et al. [88] reported an A1-D-A2-structured COF photocatalyst for ciprofloxacin degradation, with Gaussian 16 B3LYP/6-31G(d) calculations showing that COF-Tp-TAPT has higher intramolecular polarity (0.018 Debye/Å^2^) than COF-Tp-TTPB (0.013 Debye/Å^2^), a broader ESP range, and a smaller S_m_ index (0.291 vs. 0.304). These descriptors correlate with the experimental rate constants (1.06 h^−1^ vs. 0.26 h^−1^), and the mechanistic interpretation—that the stronger internal electric field promotes charge separation—is supported by the KPFM, zeta potential, PL, and photocurrent measurements. The DFT contribution consists of qualitative electronic-structure descriptors rather than adsorption energetics, and the study does not report ΔG for the degradation steps. The correlation between the computed polarity descriptors and the measured rate constants is a structure–activity relationship based on two materials and should therefore be tested across a larger series before being generalized.

Liu et al. [87] combined DFT with UPS and XPS for cellulose nanocrystal–COF S-scheme heterojunctions, reporting work functions of 4.41, 3.56, 3.33, and 3.83 eV for C-CNC, Br-COF, OH-COF, and OMe-COF, respectively. The band alignment analysis shows that the C-CNC and X-COF energy levels satisfy the S-scheme criteria, while the electron density difference and charge displacement profiles indicate interfacial charge redistribution. The mechanistic conclusion—that the built-in electric field enables spatial carrier separation and increases O_2_^−^ generation—is supported by the radical-trapping and EPR experiments, and the rate enhancement (15.7× over C-CNC, 1.6× over Br-COF) is consistent with the proposed mechanism. The DFT results provide qualitative descriptors of band alignment and charge redistribution.

### 5.8. Experimental Benchmarks for Adsorption Mechanism and Kinetics

While DFT provides atomic-level mechanistic insight, interpretation of computed binding energies and proposed adsorption mechanisms benefits from comparison with well-characterized experimental systems in which the dominant interaction type and kinetic regime have been established. Two recent studies on chemically modified polysaccharide sorbents illustrate this comparison. Farid-ul-Haq et al. [20] synthesized the sodium salt of succinylated *Artemisia vulgaris* mucilage (SSAVH) and demonstrated that Cd^2+^ uptake proceeded through an ion-exchange mechanism, achieving a Langmuir maximum sorption capacity of 263.15 mg g^−1^ from distilled water and 243.90 mg g^−1^ from spiked high-hardness groundwater. The experimental data fitted the pseudo-second-order kinetic model (R^2^ > 0.99), and the Dubinin–Radushkevich mean sorption energy of 11.96 kcal mol^−1^ indicated chemisorption as the rate-determining step. Similarly, Khan et al. [21] reported that the sodic form of maleated hydroxyethyl cellulose (HEC-MAL-Na) removed Cd(II) with a maximum capacity of 277.7 mg g^−1^ at pH 5.5. The ion-exchange mechanism was evaluated using Boyd’s rate equation and the pseudo-second-order model, while regeneration over five cycles resulted in only a marginal decrease in capacity. These studies provide an experimental set of characteristics for Cd^2+^ chemisorption on carboxylate-functionalized sorbents, including ion exchange, pseudo-second-order kinetics, exothermic thermodynamics (negative ΔH° and ΔG°), and negative ΔS°, indicating adsorbate–adsorbent interactions. Similar mechanistic signatures have been reported for Cd^2+^, Pb^2+^, and Hg^2+^ on carboxylate- and thiol-functionalized COFs discussed in Section 4 [22,24,26,35], where DFT-calculated binding energies in the chemisorption range (−0.9 to −3.0 eV) and charge-transfer magnitudes (0.5–0.9 e) are consistent with the ion-exchange and coordination mechanisms inferred experimentally. This correspondence between DFT-derived descriptors and experimental kinetic and thermodynamic characteristics provides a basis for using computed adsorption energies as descriptors in COF sorbent design. Nevertheless, as discussed in Section 2 and Section 7, direct quantitative mapping between DFT binding energies and experimentally measured capacities remains complicated by solvation, pH-dependent speciation, and competing-ion effects that are not captured in standard static DFT calculations [20,21].

Table 3 summarizes the DFT studies on organic pollutant removal using COF-based adsorbents, providing a comprehensive overview of the systems studied, computational methods employed, adsorption energies, mechanisms, and key findings.

## 6. DFT Studies on Photocatalytic Degradation

While Section 3, Section 4 and Section 5 addressed adsorptive capture as a contaminant removal mechanism, photocatalytic transformation constitutes a mechanistically distinct process in which COFs absorb light, generate charge carriers, and drive redox reactions that degrade or mineralize pollutants.

### 6.1. Azole-Linked COFs: Linkage Microenvironment Effects

Mou et al. [89] conducted a systematic DFT investigation of linkage chemistry effects on photocatalytic H_2_O_2_ production, comparing TZ-COF, OZ-COF, and IZ-COF frameworks. Using the DMol3 program with the PBE functional and COSMO solvation model, they calculated HOMO–LUMO distributions, density of states, and Gibbs free-energy diagrams for both the 2e^−^ oxygen reduction reaction (ORR) and 4e^−^ water oxidation reaction (WOR). The calculations showed that TZ-COF had HOMO orbitals localized on the pyrene donor unit and LUMO orbitals at the pyrene–azole interface, consistent with a donor–π–acceptor (D–π–A) design. The calculated Gibbs free-energy barrier for the rate-determining *OOH formation step was 0.61 eV for TZ-COF, compared to 0.91 eV for OZ-COF and 0.82 eV for IZ-COF. For the WOR pathway, TZ-COF had a minimum Gibbs free-energy barrier of 2.15 eV for the rate-limiting step. The experimental H_2_O_2_ production rates followed the order TZ-COF (268 μmol h^−1^ g^−1^) > OZ-COF (220 μmol h^−1^ g^−1^) > IZ-COF (102 μmol h^−1^ g^−1^), consistent with the DFT-predicted trend. However, these Gibbs free-energy values were calculated for idealized periodic frameworks without explicit solvation of the reaction intermediates, and the absolute values should therefore not be compared directly with experimental activation energies.

### 6.2. Cyanide-Based COFs: Yeager-Type Adsorption and Enhanced ORR

Zhou et al. [90] employed DFT calculations to investigate cyanide-based TBTN-COF for overall photocatalytic H_2_O_2_ production, comparing it with triazine-based TMT-COF. Using the DMol3 program with the PBE/DND level and COSMO solvent model, they calculated electronic structures and free-energy diagrams for the 2e^−^ ORR pathway. The calculations revealed that TBTN-COF demonstrated a lower free energy for the rate-determining *OOH formation step ($\Delta G_{\mathrm{RDS}}=0.6$ eV) compared to TMT-COF (0.75 eV). The DFT analysis showed that the cyanide active site enables Yeager-type (bridged) adsorption of *O_2_^−^ intermediates rather than the Pauling-type (end-on) adsorption observed for triazine active sites. This adsorption mode, involving dual binding sites with concentrated electron density, reduces the energy barrier for *OOH formation. The calculated electron distribution revealed that TBTN active sites possess double binding sites with concentrated electron density, facilitating bridged *O_2_^−^ formation, whereas TMT active sites show only single binding sites, limiting Yeager-type adsorption. The formation of *OOH on cyanide-rich TBTN had a highly negative ΔG of −1.81 eV, compared to −0.79 eV for TMT. Experimentally, TBTN-COF achieved a H_2_O_2_ yield rate of 11,013 μmol h^−1^ g^−1^ without sacrificial agents, approximately twice that of TMT-COF (6392 μmol h^−1^ g^−1^). The lower ORR barrier predicted by DFT is consistent with the higher experimental production rate and supports the proposed role of cyanide groups, although the 1.7-fold difference in experimental rates is smaller than the 2.3-fold difference in $\Delta G_{\mathrm{RDS}}$ values, indicating that other factors, such as light absorption and charge separation efficiency, also influence the photocatalytic rate.

### 6.3. β-Ketoenamine COFs: Structure–Activity Relationships with Antibiotics

Wang et al. [91] utilized DFT calculations to investigate β-ketoenamine COFs with varying diamine linking blocks (Tp-PA-COF, Tp-DPA-COF, Tp-TPA-COF, and Tp-QPA-COF) for simultaneous in situ H_2_O_2_ photosynthesis and antibiotic wastewater degradation. The calculations employed the Gaussian 09 package with the B3LYP functional and 6-31G(d) basis set, incorporating D3 dispersion corrections. Molecular orbital energy calculations demonstrated that antibiotics with higher $E_{\mathrm{HOMO}}$ and lower vertical ionization potential (VIP) values, particularly TC (−6.4156 eV) and OTC (−6.4137 eV), exhibited greater electron-donating capability. The HOMO of TC and OTC showed the smallest energy gap with the LUMO of the catalyst, enabling electron transfer from the antibiotics to the COF structure and subsequent participation in oxygen reduction. The DFT calculations indicated that antibiotics containing electron-donating groups (EDGs) enhanced H_2_O_2_ production and degradation efficiency. Experimentally, Tp-QPA-COF achieved a H_2_O_2_ production rate of 4.16 mM h^−1^ g^−1^ with 81.5% TC removal efficiency. However, the DFT calculations were performed using cluster models rather than periodic frameworks. The correlation between the computed $E_{\mathrm{HOMO}}$ values and experimental degradation rate constants (k) was strong for most antibiotics, but the hydrochloride salt forms deviated significantly, which was attributed to protonation effects not captured in the calculations.

### 6.4. Fluorinated Triazine COFs: Dual ORR and WOR Pathways

Jin et al. [92] performed DFT calculations on partially fluorinated triazine-based COFs (TP/TAPT-F COF) for photocatalytic H_2_O_2_ production. Using the DMol3 program with the PBE functional and DFT-D correction, they calculated projected density of states (PDOS), adsorption energies, and Gibbs free-energy diagrams. The PDOS analysis revealed that the valence and conduction bands are primarily composed of N 2p and C 2p orbitals, respectively, identifying N and C as the dominant oxidation and reduction centers. The N 2p states in TP/TAPT-F COF occupy an intermediate energy position relative to TP/TAPT and TAPT-F COFs, which is associated with bonding interactions and photocatalytic activity. The charge density difference analysis revealed that H_2_O at the top position loses more electrons, indicating that 4e^−^ WOR preferentially occurs at the top position, while 2e^−^ ORR tends to occur at the edges of the nanosheets. The Gibbs free-energy diagrams showed that TP/TAPT-F COF had a lower ΔG for the rate-determining *OOH formation step than TP/TAPT and TAPT-F COFs. For the WOR step, TP/TAPT-F COF showed a low energy barrier for the *OH → *O oxidation step, which is associated with O_2_ evolution. Experimentally, TP/TAPT-F COF achieved a H_2_O_2_ production rate of 1655 μmol h^−1^ g^−1^ with 83.8% selectivity, compared with 797 μmol h^−1^ g^−1^ for TP/TAPT and 185 μmol h^−1^ g^−1^ for TAPT-F. DFT calculations employed periodic models with implicit solvation (COSMO), whereas the reported ΔG values do not include zero-point energy corrections or explicit treatment of the aqueous phase.

### 6.5. S-Scheme Heterojunctions: Interfacial Charge Transfer

Xu et al. [93] developed an S-scheme heterojunction photocatalyst (Tph-Dha-COF@Nb_2_C) for photocatalytic H_2_O_2_ production via the 2e^−^ ORR. DFT calculations using the DMol3 program at the PBE/DND level were employed to investigate adsorption and reaction mechanisms. The adsorption energy calculations showed that O_2_ adsorption at the interface between the imine bond and Nb_2_C-NH_2_ was stronger ($E_{\mathrm{ads}}=-0.82$ eV) than at pyrrolic nitrogen sites ($E_{\mathrm{ads}}=-0.15$ eV), indicating that the heterojunction interface provides a favorable site for O_2_ adsorption. Gibbs free-energy calculations for the two-step ORR pathway showed ΔG values for *OOH formation of −0.11, −0.19, and −0.28 eV for Nb_2_C-NH_2_, Tph-Dha-COF, and Tph-Dha-COF@Nb_2_C, respectively. The heterojunction reduced the ΔG barrier for *OOH formation and yielded a lower ΔG for *HOOOH formation (−1.27 eV) than Nb_2_C-NH_2_ (−1.15 eV) and Tph-Dha-COF (−1.08 eV). Work-function calculations based on UPS spectra gave Fermi levels of 3.42 eV for Nb_2_C-NH_2_, 4.21 eV for Tph-Dha-COF, and 3.97 eV for Tph-Dha-COF@Nb_2_C, supporting the formation of an internal electric field at the heterojunction interface. In situ XPS measurements were consistent with the DFT results, showing negative shifts in the Nb 3d binding energies under visible-light irradiation, which were assigned to increased electron density at Nb sites. Experimentally, Tph-Dha-COF@Nb_2_C achieved a H_2_O_2_ production rate of 1833 μmol g^−1^ h^−1^ without sacrificial agents. The DFT calculations were performed using periodic models; however, the reported ΔG values represent electronic energies without thermal corrections, and the solvation treatment was not explicitly specified.

### 6.6. Ti-Oxo Cluster Confined COFs: Photocatalytic Uranium Reduction

Zhang et al. [94] employed DFT calculations to investigate a Ti-oxo cluster-confined COF (TiOCs/COF-TZ) for photocatalytic uranium reduction from seawater. Using the CASTEP program with the PBE functional and ultrasoft pseudopotentials, they calculated the electronic structures of COF-TZ and TiOCs/COF-TZ. The calculations showed that the introduction of TiOCs, including through covalent confinement, causes little change in the electronic structure of the parent COF-TZ, with comparable HOMO–LUMO gaps of 1.44 eV for COF-TZ and 1.53 eV for TiOCs/COF-TZ. However, the presence of TiOCs was associated with photocatalytic reduction of UO_2_(CO_3_)_3_^4−^. Geometry optimization showed that two CO_3_^2−^ ions can simultaneously coordinate with two Ti^4+^ centers by sharing four O atoms (ΔG=−12.18 kcal/mol). Following Ti–CO_3_^2−^ coordination, the U–CO_3_^2−^ bond length near the titanium centers increased, while the U=O bond length decreased, consistent with Ti^4+^–CO_3_^2−^ coordination. The LUMO of UO_2_(CO_3_)_3_^4−^/TiOCs/COF-TZ contained major U(5f) and minor Ti(3d) contributions, with the LUMO energy decreasing from −2.58 to −3.80 eV. This electronic structure permits electron transfer from ETTA to U(5f) through a long-range charge-transfer pathway. Experimentally, TiOCs/COF-TZ achieved approximately 89.9% U(VI) extraction from natural seawater under visible-light irradiation, compared with approximately 36.4% under dark conditions. DFT calculations employed periodic models with the PBE functional; however, the reported values correspond to gas-phase reactions without solvation, and relativistic effects for uranium were treated only at the scalar-relativistic level. 

### 6.7. Mass Transfer Gating: Sacrificial Agent Effects

Xue et al. [95] conducted DFT calculations on benzothiazole-based COFs (Tp-BTz COF and Tp-TTz COF) to investigate the sacrificial-agent-triggered mass transfer gating (MTG) strategy. Using the DMol3 program with the PBE functional and DFT-D correction, they calculated the O_2_-binding characteristics of secondary N, N, and S active sites. The calculations showed that the S sites in both Tp-BTz COF and Tp-TTz COF had lower O_2_ adsorption energies than the N and secondary N sites. Interfragment charge-transfer analysis showed electron-density redistribution at the benzothiazole-sulfur active sites during O_2_ adsorption, with Tp-TTz COF exhibiting higher interfacial charge transfer (0.93 e^−^) than Tp-BTz COF (0.86 e^−^). BA adsorption energies were more negative for Tp-BTz COF ($E_{\mathrm{ads}}=-$1.65 eV) than for Tp-TTz COF ($E_{\mathrm{ads}}=-$ 0.30 eV), and this difference was proposed to account for the different effects of BA on O_2_ adsorption. Gibbs free-energy diagrams showed that the first PCET step (O_2_ → *OOH) is the rate-determining step for both COFs (ΔG=0.63 eV for Tp-BTz COF and 0.66 eV for Tp-TTz COF). In the presence of BA as a sacrificial agent, the free-energy barrier for this step decreased to 0.11 eV for Tp-BTz COF and 0.36 eV for Tp-TTz COF, while the subsequent steps proceeded with decreasing free energy. These calculations support the proposed role of BA as a proton donor in the 2e^−^ ORR pathway. Experimentally, Tp-BTz COF achieved a H_2_O_2_ production rate of 100.9 mmol g^−1^ h^−1^ in a water/BA (9:1) system, compared with 1.8 times lower production for Tp-TTz COF. The DFT calculations included dispersion corrections but employed implicit solvation models that may not fully represent the biphasic water/BA environment.

### 6.8. COF/g-C_3_N_4_ Heterojunctions: Type-II Charge Transfer

Hassan et al. [96] employed DFT calculations to investigate BT-Py COF/g-C_3_N_4_ heterojunction photocatalysts for H_2_ production. Using the DMol3 program with the GGA-PBE functional and DFT-D correction, they calculated adsorption energies, HOMO–LUMO distributions, and chemical reactivity descriptors. The $E_{\mathrm{ads}}$ value between COF and g-C_3_N_4_ was calculated to be −2.40 eV, corresponding to exothermic π–π stacking interactions between the two components. The calculated band gaps of COF and CN were 1.31 and 2.05 eV, respectively, whereas the COF/CN heterostructure exhibited a band gap of 0.48 eV, which the authors attributed to interfacial coupling and charge redistribution. HOMO–LUMO analysis indicated electron transfer from the COF LUMO toward CN, while hole transfer occurred from the CN HOMO toward COF, supporting the proposed type-II heterojunction configuration. Chemical reactivity descriptors for COF/CN included a band gap of 0.48 eV, low ionization potential, high electronegativity, an electrophilicity index of 2.80 eV, and charge transfer, which were associated with the calculated electronic structure relevant to photocatalytic HER. Experimentally, the 30% COF/CN heterojunction achieved a hydrogen evolution rate of 27,540 μmol g^−1^ h^−1^ with an apparent quantum yield of 15.5% at 420 nm. The DFT calculations employed cluster models rather than periodic frameworks, and the calculated band gap of 0.48 eV is substantially lower than typical experimental values reported for such heterojunctions, consistent with the known tendency of GGA functionals to underestimate band gaps.

Table 4 summarizes the DFT studies on photocatalytic degradation processes using COF-based adsorbents, providing a comprehensive overview of the systems studied, computational methods employed, energies, mechanisms, and key findings.

## 7. Challenges and Future Perspectives

Despite the progress in DFT-based investigations of COFs for environmental remediation, several challenges remain that limit the translation of computational insights into practical applications. The methodological limitations underlying these challenges—including functional dependence, dispersion treatment, solvation and pH, structural idealization, dynamic framework behavior, periodic versus cluster models, and the gap between 0 K calculations and ambient conditions—are examined in detail in Section 2.3. The present section therefore focuses not on reiterating these limitations but on the strategies, standards, and emerging directions that may help address them and advance the field toward more reliable and application-relevant computational studies.

**Figure 6:**
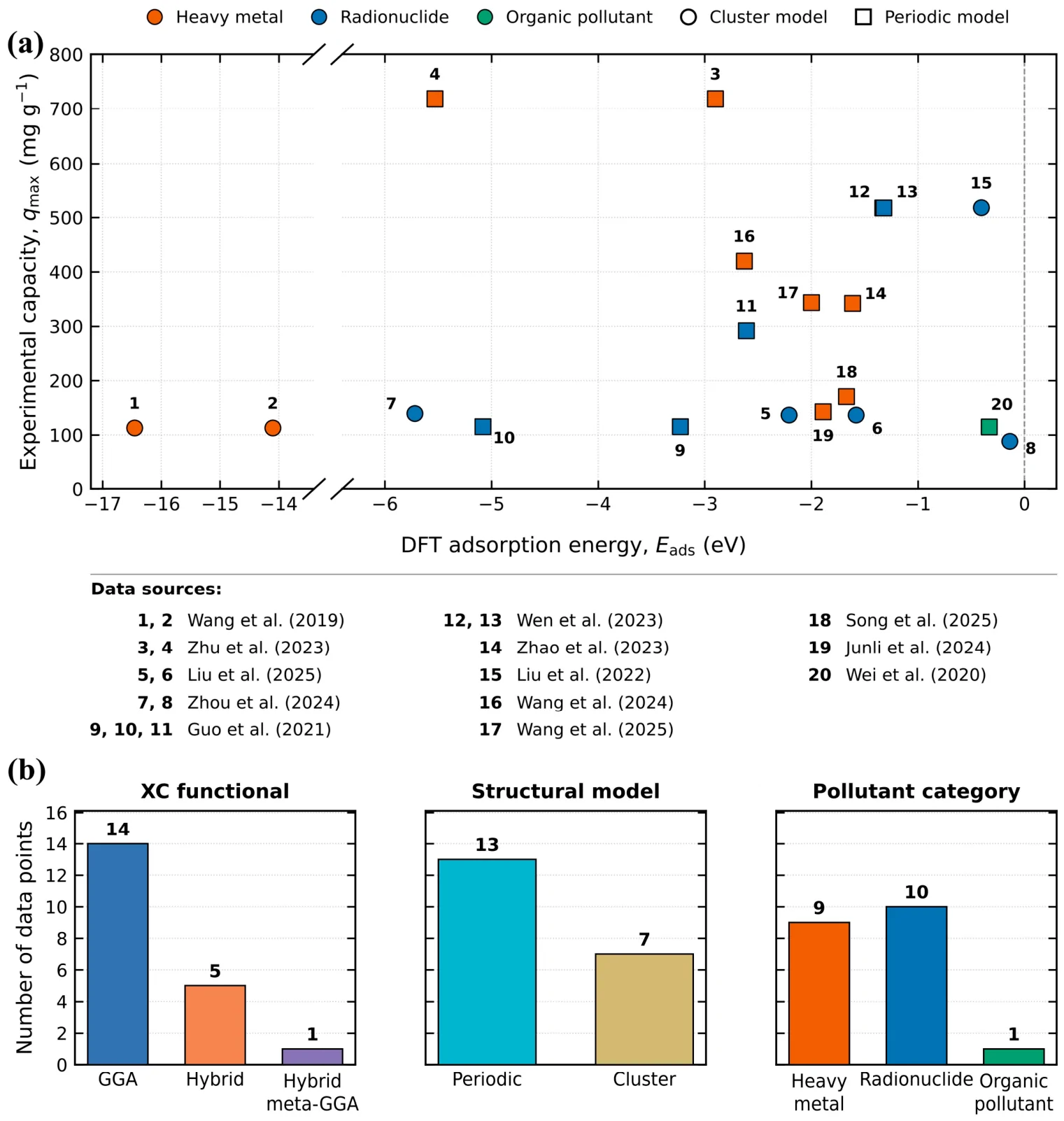
Comparison of adsorption energies versus experimental capacities (for selected 20 data points, namely data 1, 2 from [23], 3, 4 from [35], 5, 6 from [40], 7, 8 from [41], 9–11 from [42], 12, 13 from [44], 14 from [46], 15 from [49], 16 from [51], 17 from [52], 18 from [53], 19 from [54], 20 from [71]), with different symbols/colors for computational methodologies. (**a**) DFT-computed adsorption energies versus experimental capacities for 20 COF systems. Colors denote pollutant class (red = heavy metals; blue = radionuclides; green = organic pollutants); markers denote model type (circle = cluster; square = periodic). (**b**) Distribution of computational methodologies across the 20 compiled data points, including functional class, model type, and pollutant category.

A recurring obstacle to cumulative progress is the lack of direct comparability across the reviewed literature, as shown in Figure 6. Reported adsorption energies vary not only because the physical systems differ but also because the computational protocols differ. To enable future comparisons to be quantitative rather than qualitative, DFT studies of COF adsorption and photocatalysis should report the following at minimum: (i) the exchange-correlation functional and whether it is GGA, meta-GGA, hybrid, or double-hybrid; (ii) the dispersion-correction scheme; (iii) the basis set or pseudopotential used for each element; (iv) whether relativistic effects are included and at what level (scalar, spin-orbit, or none); (v) whether the model is periodic or cluster, and, for periodic calculations, the cell dimensions, k-point sampling, and stacking arrangement; (vi) the solvation model and, where relevant, the modeled pH and chemical speciation; (vii) whether the reported energy is E_ads_ or ΔG_ads_, with explicit specification of the reference state; and (viii) whether counterpoise or basis-set superposition error corrections were applied. Standardization of these parameters would facilitate the construction of comparable datasets and enable quantitative meta-analysis rather than primarily qualitative cross-study comparison.

Environmental remediation involves phenomena spanning multiple length and time scales, from atomic-scale binding interactions to macroscopic mass transport in porous COF membranes [97]. While DFT provides atomistic information on electronic structure and chemical interactions, it cannot by itself capture diffusion kinetics, membrane permeability, or long-range electrostatic effects over experimentally relevant scales. Multi-scale modeling frameworks that combine DFT with molecular dynamics, Monte Carlo simulations, and continuum models are therefore needed to bridge these scales [98,99,100,101]. Such frameworks could connect atomic-level binding predictions with device-relevant performance metrics, thereby linking mechanistic calculations with transport and engineering considerations.

Several emerging approaches may help address the limitations identified above. Machine-learned force fields trained against DFT data are beginning to enable simulations of dynamic adsorption processes over longer molecular-dynamics timescales [102], addressing the limited treatment of framework flexibility discussed in Section 2.3. The integration of DFT with operando spectroscopy and in situ characterization can provide experimental tests of computational predictions under working conditions rather than relying exclusively on idealized structural models [103]. Hierarchical COF structures that combine microporosity for selective binding with mesoporosity for mass transport provide another area in which DFT calculations may be integrated with experimental synthesis and transport studies [98,102,103,104,105,106,107,108]. Extending DFT investigations to emerging pollutants such as microplastics and antibiotic resistance genes could further broaden the range of environmental systems considered in COF-based remediation research.

Taken together, these limitations define the interpretive boundary of the present review. The mechanistic findings identify relationships between functional groups and contaminants, the associated interaction types, and charge-transfer characteristics. These qualitative features can provide mechanistic information even when methodological differences affect the corresponding numerical values. In contrast, numerical rankings of adsorption energies across studies and their direct translation into expected adsorption capacities are not justified without accounting for differences in computational methodology and experimental conditions. Addressing this gap will require consistent reporting of the computational parameters outlined above, together with multi-scale frameworks that connect atomic-level calculations to experimentally accessible quantities. Such integration can improve the reproducibility and quantitative interpretation of DFT-guided COF studies and facilitate their application to water treatment and other environmental remediation systems.

## 8. Conclusions

This review evaluated DFT studies on the adsorption and photocatalytic activity of COFs for environmental remediation. The surveyed literature demonstrates that DFT has become the primary tool for resolving the atomic-level interactions that govern contaminant capture, yielding binding energies, charge-transfer pathways, and electronic-structure modifications that are difficult to access experimentally.

Across the contaminant classes examined, a consistent structure–property picture emerges. For heavy metals, soft donor atoms (S, N) in thiol, thioether, and thiazole functionalities form strong chelation complexes with Hg^2+^, Pb^2+^, Cd^2+^, and Au^3+^, whereas hard donors such as carboxyl and phosphoryl oxygen govern uranyl binding. Radionuclide sequestration by amidoxime- and phosphonate-functionalized COFs is driven by multidentate coordination, with photocatalytic reduction providing an additional immobilization pathway for U(VI). Organic pollutant removal depends on a different set of interactions, including π–π stacking, hydrophobic association, and hydrogen and halogen bonding, where pore-size engineering determines selectivity. Photocatalytic applications, examined through band-structure and reaction-energy analysis, show that linkage chemistry, fluorination, and heterojunction formation control charge separation and lower the barriers for H_2_O_2_ production and pollutant degradation. These findings collectively confirm that the functional-group and pore-architecture descriptors extracted from DFT are transferable predictors of COF performance across chemically distinct contaminant classes.

Moreover, as discussed in Section 2 and Section 7, the reviewed studies vary widely in functional choice, dispersion treatment, solvation modeling, and whether electronic or Gibbs adsorption energies are reported, which restricts quantitative cross-comparison and others. Most calculations assume idealized, defect-free frameworks at 0 K in implicit solvents, whereas experimental adsorption occurs at ambient conditions with pH-dependent speciation, competing ions, and structural defects. The photocatalytic studies, while mechanistically informative, frequently rely on single-particle band alignments rather than explicit excited-state or non-adiabatic treatments. These gaps mean that DFT-derived rankings should be treated as mechanistic guidance rather than direct predictions of experimental capacity.

The most immediate translational opportunity identified in this review is the assembly of the reported descriptors, frontier-orbital localization, band-edge positions, interfacial charge-transfer magnitudes, and adsorption free energies of reaction intermediates, into a unified, FAIR-compliant dataset. Such a dataset would connect directly to data-driven photocatalyst and adsorbent design workflows, including generative architectures and multi-objective optimization, and would allow the vast COF chemical space to be searched against combined stability, cost, and activity criteria. Closing the loop between DFT prediction, targeted synthesis, spectroscopic characterization, and standardized activity testing remains the decisive step for converting mechanistic understanding into validated materials. Herein, the DFT-supported design cycle offers a realistic route toward scalable COF-based water-purification and other environmental remediation technologies.

## Figures and Tables

**Table 1:** Summary of DFT studies on heavy metal adsorption on COFs.

DFT Model/System	Method	Energy, Free Energy, and Others	Adsorption Mechanism	Key Findings
Hg^2+^@Boroxine [22]	HSE06/6-311+g(d)//LANL2DZ; Gaussian 09; PCM solvent	$E_{\mathrm{binding}}=$ −32.14 kcal/mol	Ionic interaction with charge transfer (0.53 e)	*E_gap_* reduced; k = 8.41 × 10^13^ s^−1^
Ag NPs@COF-LZU1/Hg [23]	PW91/BS-II & M06-2X/BS-I; DFT-D3; NBO analysis	$E_{\mathrm{ads}}$ is −325.22 kcal/mol (for PW91), and −379.50 kcal/mol (for M06-2X)	Reduction and Ag-Hg amalgam formation	Ag atom utilization 150%; multi-step exothermic process (−0.96 eV)
COF_ETA-BPT-S,N/Hg^2+^ [24]	B3LYP/SDD; Gaussian 16; MBO & IRI analysis	MBO analysis gave 0.5025 for Hg–S and 0.4310 for Hg–N (most stable configurations).	Coordination + electrostatic + vdW, coordination bonds (S-Hg: 0.5025 MBO), notable attraction + vdW interactions	Ultrafast removal (<10s) to 0.28 μg/L; $q_{\max}\;$ = 621.65 mg/g, stronger coordination with S than N; chemisorption dominant, weaker interaction than Hg^2+^ and Hg(OH)^+^
MCM/Hg^2+^ [25]	M06-2X/6-31G(d,p)//LANL2DZ	$E_{\mathrm{binding}}=$ 38.94 kcal/mol (Schiff base-Hg)	Chelation + electrostatic binding	$q_{\max}$ = 843.89 mg/g; LOD = 0.01 μg/L; good recyclability
COF@HPLC/Pb^2+^ [26]	B3LYP/SDD; Gaussian 09; PCM solvent	N3 site gave the shortest Pb–N distance (2.31 Å)	Chelation + electrostatic interaction	N3 site strongest; S2 weakest; $q_{\max}$ = 251.37 mg/g
Cr_2_O_7_^2−^@Imi-TPP-COF-Br-AR [27]	M06-2X-D3/def2-SVP (opt); M06-2X-D3/def2-TZVPD (SP); Gaussian 09; RDG analysis	$E_{\mathrm{interaction}}=$ −20 kcal/mol (difference between modes)	Electrostatic + H-bonding (N-H⋯O, C-H⋯O) + vdW	Imidazole ring primary binding site; $q_{\max}$ = 373.14 mg/g; LOD = 41 nM
As(III)@DU66 [28]	M06-L/LANL2DZ(Zr,As)//6-31G(d,p); EXAFS fitting	Mulliken charge of arsenic ranged from 1.25 to 1.29	Covalent bonding (Zr-O-As)	Model 3 best fit; As-O = 1.71–1.72 Å; $q_{\max}$ = 204 mg/g at pH 8
PBCOF/Pb^2+^ [29]	B3LYP/6-31G; Gaussian 09; TD-DFT/CAM-B3LYP/LANL2DZ	A Pb charge of 0.411 (from NBO), indicating electron donation from the cyano group	Coordination with CN groups (6S,6p orbitals)	Shortest M-L bonds (2.161–3.372 Å); largest red shift to 510 nm
sTPA-PZ/Cl^−^ [30]	GGA-PBE/Dmol3; Materials Studio 2020	$E_{\mathrm{binding}}$ = −0.99 eV	Electrostatic + size sieving	Cl^−^ diffusion: 0.20 Å^2^ ps^−1^; Na_2_SO_4_ rejection 99.1%
sTPA-PZ/SO_4_^2−^ [30]	GGA-PBE/Dmol3; Materials Studio 2020	$E_{\mathrm{binding}}$ = 9.2 eV	Electrostatic repulsion + size sieving	Energy barrier 455.5 kJ/mol; Cl^−^/SO_4_^2−^ selectivity 66
COF-GO-50/M^n+^ [31]	First-principles DFT	For carbonyl oxygen complexation with metals, E_ads_ is −1.947 eV for Pb^2+^, −1.182 eV for Ni^2+^, −0.791 eV for Co^2+^, −0.769 eV for Cu^2+^, −0.086 eV for Cd^2+^, −1.429 eV for Fe^3+^, and −1.22 eV for Cr^3+^	Central pore + carbonyl O complexation	Removal: Cu^2+^/Fe^3+^ (98%), Cr^3+^ (97%), Pb^2+^ (96%), others (>93%); carbonyl O binding dominates; divalent ions stronger than trivalent; Cd^2+^ weakest
TTASDFP/Au^3+^ [32]	DFT calculations using VASP with PBE-GGA functional and PAW pseudopotentials; XPS analysis; HSAB principle	Strong Au-S binding (no specific E_ads_ value reported in main text)	Chemisorption via Au-S chelation; XPS showed S 2p signal decrease by ~1 eV post-adsorption; Au 4f peaks at 85.1 and 88.8 eV confirmed Au^3+^	>99% removal of 9 ppm Au^3+^ in 30 s; $q_{\max}$ = 245 mg/g; selective over Cd^2+^, Co^2+^, Cu^2+^, Li^+^, Zn^2+^; captures Au^3+^ at 1–16 ppb; >99% removal after 5 cycles;
AuCl_3_@TzDa-COF [33]	PBE-GGA/PAW; VASP	$E_{\mathrm{binding}}=$ −2.32 eV	Coordination + reduction to Au(0)	$q_{\max}$ = 1866 mg/g; 99% removal in 30 min; fast kinetics
Au^3+^@UiO-66-TA [34]	B3LYP/LANL2DZ(Au)//6-31+G(d,p)	$E_{\mathrm{binding}}=$ −36.20 eV for binding of Au to S atom	Chelation + ion exchange	Au-S bond (2.38–2.39 Å); $q_{\max}$ = 374 mg/g; selectivity for Au^3+^
Bpy-sp^2^c-COF/M^2+^ [35]	GGA-PBE/Dmol3; Materials Studio	For Hg^2+^, monolayer E_ads_ values were −2.27 eV (monodentate, N site) and −2.90 eV (bidentate, N=N site); double-layer configurations gave −3.58 and −5.53 eV	Coordination with N sites	Electronegativity order Hg^2+^ (2.00) > Ni^2+^ (1.92) > Cu^2+^ (1.90) > Co^2+^ (1.88)
Tu@COF-NH_2_/AuCl_4_^−^ [36]	Gaussian 16; ESP analysis	$E_{\mathrm{binding}}=$ −16.01 eV (Model 1)	Electrostatic + coordination + reduction	$q_{\max}$ = 2002.7 mg/g; 99.99% selectivity; 4 cycles stability
H_2_Cr_2_O_7_@COF-PDZ [37]	GGA-PBE/PAW; VASP; GGA+U (U = 2.5 eV)	$E_{\mathrm{ads}}$ = −26.65 kJ/mol	Electron donor-acceptor (EDA)	Best among 5 COFs; k = 0.141 min^−1^; 99% removal
H_2_Cr_2_O_7_@COF-PMD [37]	GGA-PBE/PAW; VASP; GGA+U (U = 2.5 eV)	$E_{\mathrm{ads}}$ = −17.37 kJ/mol	EDA	Second-best; k = 0.028 min^−1^
H_2_Cr_2_O_7_@COF-PZ [37]	GGA-PBE/PAW; VASP; GGA+U (U = 2.5 eV)	$E_{\mathrm{ads}}$ = −2.53 kJ/mol	Weak EDA interaction	Poor adsorption; k = 0.011 min^−1^
CrO_4_^2−^@BPTP Foam [38]	B3LYP/def2-TZVP; DFT-D3; Gaussian 16	$E_{\mathrm{binding}}=$ −17.76 eV	Non-covalent + hydrogen bonding	$\;q_{\max}$ = 448.09 mg/g; 98.8% removal in 3 min

**Table 2:** Summary of DFT studies on radionuclide adsorption on COFs.

DFT Model/System	Method	Energy, Free Energy, and Others	Adsorption Mechanism	Key Findings
Tp-PDAN-AO/UO_2_^2+^ [39]	PBE0-D3/6-31G(d)/SDD; Gaussian 16; SMD solvation	ΔG = −0.585 kcal/mol (Mode III)	η^2^ coordination (N,O chelation)	$q_{\max}$ = 757.62 mg/g (photo); 6 coordination modes identified
LZU-COF-COOH/UO_2_^2+^ [40]	PBE0-D3/6-31G(d,p)/SDD; Gaussian	$E_{\mathrm{ads}}$ = −2.21 eV (LZU-COF-COOH); $E_{\mathrm{ads}}$ = −1.58 eV (LZU-COF)	Surface complexation + electrostatic	Smaller bond lengths/angles; $q_{\max}$ = 136.7 mg/g; 1 h equilibrium
TzDva-COOH/UO_2_^2+^ (Zhou et al., 2024) [41]	B3LYP/6-31G(d,p)	$E_{\mathrm{ads}}$ = −5.72 eV	Chelation + electrostatic	U-O bond 2.37 Å; $q_{\max}$ = 139.5 mg/g
TzDva-NH_2_/UO_2_^2+^ (Zhou et al., 2024) [41]	B3LYP/6-31G(d,p)	$E_{\mathrm{ads}}$ = −0.14 eV	Chelation + electrostatic	U-N bond 2.04 Å; $q_{\max}$ = 88.3 mg/g
TpBpy/UO_2_^2+^ (Guo et al., 2021) [42]	PBE-GGA/VASP	$E_{\mathrm{ads}}$ = −3.23 eV (N site, monolayer); $E_{\mathrm{ads}}$ = −5.08 eV (N site, interlayer)	Chelation (N,N bidentate)	Double N structure enhances chelation; $q_{\max}$ = 115.45 mg/g
TpPy/UO_2_^2+^ (Guo et al., 2021) [42]	PBE-GGA/VASP	$E_{\mathrm{ads}}$ = −2.61 eV (N=O site)	Coordination (O,N)	Charge transfer 0.84 e; $q_{\max}$ = 291.79 mg/g
TpDBD-Phos/UO_2_^2+^ (Mei & Yan, 2023) [43]	PBE0/Gaussian 16	Stronger than TpPa-2OH-Phos	Synergistic phosphonic + benzoxazole	$q_{\max}$ = 633.3 mg/g; excellent selectivity
DQTP COF/UO_2_^2+^ (Wen et al., 2023) [44]	PBE-GGA/VASP	$E_{\mathrm{ads}}$ = −1.33 eV (β-ketoenamine); $E_{\mathrm{ads}}$ = −1.32 eV (quinone)	Coordination, electrostatic	U-O 2.37 Å; charge transfer 0.17 e; $q_{\max}$ = 517.62 mg/g
Tp-Db/UO_2_^2+^ (Liu et al., 2025) [45]	PBE-GGA/VASP	UO_2_^2+^ preferentially coordinates with the carbonyl group in Tp-Db	Carbonyl coordination	U-O 2.26 Å; LOD = 99.34 nM; 10 s response
Tp-DGCl/Pd^2+^ (Zhao et al., 2023) [46]	PBE-GGA/DMol3	$E_{\mathrm{ads}}$ = −37.24 kcal/mol (Model-3)	Electrostatic + chelation	$q_{\max}$ = 342.1 mg/g; excellent selectivity
NCOF-3/UO_2_^2+^ (He et al., 2025) [47]	Materials Studio/DFT	Reduced exciton binding	Photocatalytic reduction	Asymmetric D′-A-D″ structure; $q_{\max}$ = 1084 mg/g
XJCOF/GO/UO_2_^2+^ (Sun et al., 2024) [48]	PBE0/SDD/Gaussian 16	Strong interaction between sulfonic acid groups and uranyl ions	Sulfonate coordination	7s/5f(U) → 2p(S,O); $q_{\max}$ = 358.8 mg/g in 10 min
CNT/COF-OH/UO_2_^2+^ (Liu et al., 2022) [49]	Multiwfn; DFT; B3LYP 6-311G(d)/SDD, Grimme’s D3BJ dispersion correction	$E_{\mathrm{ads}}$ = −24.22 kJ/mol (COF-OH); $E_{\mathrm{ads}}$ = −39.17 kJ/mol (CNT/COF-OH)	Electrostatic reduction, complexation	Reduction potential: −0.58 V; 63.3% enhanced reduction; $q_{\max}$ *_x_* = 518.2 mg/g
DBBT/TcO_4_^−^ (Zhao et al., 2022) [50]	PBE-GGA/DFT-D3/VASP	ESP max value was 95.69 kcal/mol	Electrostatic interaction	Best among 3 cationic COFs; AA > AB stacking
COF-SSH/Pd^2+^ (Wang et al., 2024) [51]	PBE-GGA/DFT-D3/VASP	$E_{\mathrm{ads}}$ = −2.63 eV (S-H); $E_{\mathrm{ads}}$ = −2.20 eV (S); $E_{\mathrm{ads}}$ = −1.65 eV (N)	Pd-S coordination	$q_{\max}$ = 420.2 mg/g; 10 min equilibrium
TAPB-BMTTPA-COF/Pd^2+^ (Wang et al., 2025) [52]	PBE-GGA/DFT-D3/VASP	$E_{\mathrm{ads}}$ = −2.00 eV (S); $E_{\mathrm{ads}}$ = −1.83 eV (N)	Pd-S coordination	$q_{\max}$ = 343.6 mg/g; excellent selectivity
COF-42/Pd^2+^ (Song et al., 2025) [53]	PBE-GGA/DFT-D3/VASP	$E_{\mathrm{ads}}$ = −1.75 eV (N); $E_{\mathrm{ads}}$ = −1.67 eV (O)	Pd-N/O coordination	$q_{\max}$ = 170.6 mg/g at 3M HNO_3_
DHTP-TPB COF/Pd^2+^ (Junli et al., 2024) [54]	PBE-GGA/DFT-D3/VASP	$E_{\mathrm{ads}}$ = −1.89 eV (N); $E_{\mathrm{ads}}$ = −1.17 eV (NH); $E_{\mathrm{ads}}$ = −0.94 eV (OH)	Pd-N coordination	$q_{\max}$ = 142.8 mg/g; 10 min equilibrium
TpPz COF/Pd^2+^ (Hu et al., 2025) [55]	ELF calculations; DFT	$E_{\mathrm{ads}}$ = −0.70 eV (Pd^0^-TpPz with active H)	Coordination, catalytic reduction	>95% Pd selectivity
COF-V/I^−^ (Tian et al., 2023) [56]	MO6-2X/def2-SVP; Gaussian 16; IGMH; Multiwfn	$E_{\mathrm{binding}}=$ −10.4 kcal/mol (vinyl groups)	Hydrogen bonding, C-H⋯I^−^ interactions	$q_{\max}$ = 437.8 mg/g; >90% removal at 181.3-1017.5 MBq

**Table 3:** Summary of DFT studies on organic pollutant removal using COF-based adsorbents.

DFT Model/System	Pollutant	Method	Energy, Free Energy, and Others	Adsorption Mechanism	Key Findings
TPB-DMTP-COF [57]	SMT	DFT-COSMO (DMol3, GGA-PBE/DNP with Grimme D3)	SMT molecules adsorbed in the pore sites of COFs	C-H···π interaction	SMT adsorbed in pore sites via C-H···π; pH-dependent electrostatic effects
COF-PRD [58]	Bisphenols (BPA, BPB, BPF, BPZ, BPAP)	Gaussian 16 (B3LYP/6-31G(d))	DFT Fukui indices identified reactive sites	Hydroxylation, electrophilic attack, ring-opening	Degradation pathway elucidated
TFPB-TgCl [59]	Naproxen (NPX)	Gaussian 09 (M062X/def2svp with D3(BJ) dispersion)	$E_{\mathrm{binding}}\;$ = −15.70 to −131.57 kcal/mol	Electrostatic, π-π interactions	IGMH identified van der Waals as primary weak interactions; ESP confirmed electrostatic role
NH_2_-MIL-125@SNW-1 [60]	Tetracycline	Materials Studio (Dmol3, GGA-BLYP)	Valence band of the MOF to O 2p and the conduction band edge to Ti 3p	Z-scheme heterojunction	3D charge density difference confirmed electron transfer from MOF to COF
NH_2_-MIL-125@TpPa-SO_3_H [61]	Fluoroquinolones (ENO, NOR, CIP, SPA)	Gaussian 09, Materials Studio 7.0 (B3LYP-D3/6-31G(d))	NBO showed 0.153 e charge transfer;	Electrostatic + H-bonding + C-H···π	$q_{\max}$ = 444.7–457.5 mg/g (Sips model); electrostatic interactions are dominant
DAAQ-TFP/COF-78/COF-OEt [62]	Pharmaceuticals (ATN, CBZ, CIF, DFC, GEM, IBU)	Gaussian 09 (B3LYP/6-31+G)	$E_{\mathrm{ads}}\;$ = −0.81 to −1.18 eV	H-bonding, van der Waals	DFT validated MD results; van der Waals primary mechanism
TAPB-DMTP COFs [63]	Macrolides (EMC, CMC, AZM, DEG)	DFT with MD	ΔG landscape minimum, −665.81 kJ/mol (DEG/F-COFs)	H-bonding + X-H···π interactions	Functionalization with OH enhanced adsorption; metadynamics revealed free-energy landscapes
Cationic COF [64]	GenX, HFPO-TA	Gaussian 09 (B3LYP/6-31G)	$E_{\mathrm{binding}}\;$ = −82.4 kcal/mol (electrostatic)	Ion exchange + electrostatic	HFPO-TA replacement of GenX requires 0.012 eV; ESP confirmed electrostatic complementation
β-Cyclodextrin COF [65]	PFOS, PFHxS, PFBS, F53B	ORCA (B97-3c/def2-mTZVP)	$E_{\mathrm{binding}}\;$ = −37.93 kcal/mol (PFOS)	H-bonding + van der Waals + hydrophobic	Binding energy order: PFOS > F53B > PFHxS > PFBS; IGM confirmed halogen bonding
Fluorinated Quaternary Ammonium COF [66]	PFOS, PFHxS	Gaussian 09 (B3LYP/6-31G with IEFPCM)	Δ$E_{\mathrm{binding}}$ = −11.0 kJ/mol for PFOS, Δ$E_{\mathrm{binding}}$ = −8.7 kJ/mol for PFHxS	Fluorophilic interaction + ion exchange	Cl release ratio: PFHxS 0.69, PFOS 0.46; DFT revealed “fluorophilicity” mechanism
Chitosan-COF [67]	PFOA, PFOS	Gaussian 16 (B3LYP/6-311G(d))	Δ$E_{\mathrm{interaction}}$ = 33.2 kJ/mol (COF-PFOA); Δ$E_{\mathrm{interaction}}$ = 23.8 kJ/mol (CS-PFOA)	Electrostatic + H-bonding + van der Waals	Quaternary amine primary adsorption site; ESP and IRI confirmed interactions
TzTPT-COF [68]	MB, TOLO, RhB, AZA, AZB	VASP (PBE-D3)	Δ$E_{\mathrm{binding}}$ from −0.79 to −1.41 eV	Electrostatic + π-π + H-bonding	Binding energy order: MB > TOLO > AZA > AZB > RhB; consistent with experimental
Cage-COF-TP [69]	Malachite Green (MG)	GCMC (COMPASS II), DFT	Q_st_: −76.03 kcal/mol (MG)	Size matching + electrostatic	MG diffusion coefficient lowest; isosteric heat highest
Ionic COF Membrane [70]	MB (cationic), NR (neutral), MO (anionic)	Materials Studio (DMol^3^)	Δ$E_{\mathrm{interaction}}$ is −0.72 eV for MB, 12.29 eV for NR, 36.82 eV for MO	Electrostatic attraction/repulsion	DFT confirmed charge-controlled separation; MB binding is exothermic
Fe_3_O_4_@TpND [71]	BPA	VASP (PBE-D3)	$E_{\mathrm{ads}}=$ −7.65 kcal/mol	H-bonding + pore trapping	Charge density difference confirmed electron transfer; MD showed aggregation effects
R-COFs (R=H, CN, NO_2_) [72]	BPA	ORCA (B3LYP/def2-SVP)	CN-COF broadest ESP range (−36.94 to 27.63 kcal/mol);	Photocatalytic (ROS generation)	Enhanced charge polarization
PyTTA-Dva-COF [73]	BPA, BPB, BPF, DCF, SMT	Gaussian 09 (ONIOM B3LYP-D3/6-31G(d):UFF)	$E_{\mathrm{ads}}$ from 53.35 to 55.86 kJ/mol (bisphenols); from 14.89 to 23.51 kJ/mol (others)	H-bonding + π-π	Bisphenols form two H-bonds; QM/MM confirmed adsorption energies
Azo-COF (ThTFB-nN=N) [74]	Aniline	Gaussian (B3LYP/6-311G(d,p))	cis-azo configuration increased dipole moment;	PET + ESP change	PET mechanism for fluorescence quenching; 303.3 mg/g (cis); 30.7 mg/g (trans)
TAPB-TPA COF [75]	Polystyrene Nanoplastics (PSNPs)	Materials Studio (DMol3, PBE-GGA/DND)	$E_{\mathrm{ads}}=\;$ −1.04 eV	Electrostatic + van der Waals	COF (ΔE = 1.982 eV) < PSNPs (ΔE = 4.189 eV); non-bonding interactions are dominant
COF1/COF2/COF3 [76]	Triphenyl Phosphate (TPhP)	VASP (GGA-PBE)	$E_{\mathrm{sorption}}\;$ from 0.045 to 0.423 eV	Hydrophobic + π-π	Optimal pore size (COF2, 2.57 nm) gave highest capacity; DFT confirmed pore size effect
Fe/COF [77]	Phytic Acid (PA), HEDP, PMG, EDTMP, DTMP, ATMP	Materials Studio (Dmol3, GGA-BLYP)	$E_{\mathrm{ads}}$ is −1.42 eV for PA; from 0.78 to −0.99 eV for others	Multidentate coordination + electrostatic	Adsorption energy order: Fe/COF > Ni/COF > Fe-Ni/COF > Zn/COF
Heteropore COF [78]	Aryl-OPEs (TMPP, RDP, etc.)	DFT calculations	$E_{\mathrm{ads}}$ from −2.8 to −16.7 eV for HCOF; from −0.6 to −5.5 eV for MS	π-π conjugation + H-bonding	Heteropore structure enables size-selective adsorption; XPS confirmed mechanisms
TAPT-DMTA-COF [79]	PBDEs (BDE28, 47, 99, 100, 154)	Materials Studio (DMol3, GGA-PBE)	$E_{\mathrm{ads}}$ from −27.01 to −48.47 kJ/mol	Halogen bonding (N-Br/O-Br)	Positive linear relation between Eads and Br content (R^2^ = 0.8927); IGM confirmed halogen bonds
COF-S-SH [80]	BTEX (B, T, E, o-X, m-X, p-X)	VASP (GGA-PBE)	−150.2 to −255.8 kJ/mol	π-π stacking + hydrophobic	Adsorption capacity order: o-X > E > p-X > m-X > T > B
PTFE-Decorated COFs [81]	BTEX (B, E, T, X)	Gaussian 09 (B3LYP/6-311++G(2d,2p))	$E_{\mathrm{ads}}$ from 0.00265 to 0.00274 MeV	Physisorption + weak chemisorption	Fe doping enhances chemisorption;
Ag@TPHH-COF [82]	4-Nitrophenol, dyes	Materials Studio	17.44 kJ/mol (E_a_)	Catalytic reduction	TOF = 4.15 min^−1^; Ag NPs (5 ± 3 nm) uniformly dispersed; N and O atoms anchor Ag^0^
TpPa-1, TpBD, TpTph [83]	SMR	VASP (PAW-PBE)	$E_{\mathrm{ads}}$ values from −0.253 to −54.31 kJ/mol	C-H···π + H-bonding	Adsorption probability analysis; pore size affects number of adsorption sites
Hz-TPFTZ@PSf/PVP MMM [84]	Dyes (BB41, MV)	Not specified	Negative surface charge enhanced cationic dye adsorption	Electrostatic + π-π	MMM with 1.0 wt% COF showed 92% dye rejection; negative surface charge enhanced cationic dye adsorption
PA-TpPa-SO_3_H/PAN [85]	Organic chloramines precursors	Gaussian 16 (B3LYP-D3BJ with mixed basis sets)	3.49 eV (barrier for TpPa-SO_3_H)	Electrostatic repulsion	Sulfonate groups increased negatively charged area; DFT confirmed electrical repulsion mechanism
IRMOF-16/HKUST-1/ZIF-8 [86]	Phenazopyridine (PHP)	Gaussian 09 (B3LYP/6-31+G*)	−1.53 eV (IRMOF-16); −1.31 eV (HKUST-1); −0.04 eV (ZIF-8)	Electrostatic + π-π stacking	PHP adsorption: IRMOF-16 (100%) > HKUST-1 (60%) > ZIF-8 (40%)
CNC@X-COF (X=Br, OH, OMe) [87]	Tetracycline (TCH), DCF, BPA, NPX, APAP	DFT with UPS/XPS	CNC@Br-COF showed 15.7× higher TCH degradation than C-CNC	S-scheme heterojunction	BIEF enables spatial carrier separation
COF-Tp-TAPT, COF-Tp-TTPB [88]	Ciprofloxacin (CIP), other ECs	Gaussian 16 (B3LYP/6-31G(d))	The photogenerated electrons were easily separated from the phenyl donor and transferred to quinone and triazine acceptors	A1-D-A2 intramolecular charge transfer	COF-Tp-TAPT (0.018 Debay/Å^2^) > COF-Tp-TTPB (0.013 Debay/Å^2^); 91.3% CIP degradation

**Table 4:** Summary of DFT studies on COF photocatalytic activity.

DFT Model/System	Method	Energies, Free Energies, and Others	Adsorption Mechanism	Key Findings
Azole-linked COFs (TZ-COF, OZ-COF, IZ-COF) [89]	DMol3, PBE/DND, COSMO solvent model	E_ads_ (O_2_ at Site 1): −2.43 eV	2e^−^ ORR → *O_2_^−^ → *OOH → H_2_O_2_; 4h^+^ WOR → O_2_ evolution	Thiazole linkage provides optimal D-π-A configuration with lowest ORR energy barrier
Cyanide-based TBTN-COF [90]	DMol3, PBE/DND, COSMO solvent model	ΔG_RDS_ (OOH): TBTN = 0.6 eV, TMT = 0.75 eV; Δ*G* (OOH): TBTN = −1.81 eV, TMT = −0.79 eV	Yeager-type adsorption at cyanide site	Cyanide groups enable Yeager-type adsorption
β-Ketoenamine COFs (Tp-PA, DPA, TPA, QPA-COF) [91]	Gaussian 09, B3LYP/6-31G(d), D3 dispersion	The HOMO of TC and OTC showed the smallest energy gap with the LUMO of the catalyst	Antibiotics with EDG provide electrons; O_2_ reduction at phenyl chain intersections	Antibiotics with higher E(HOMO) and lower VIP enhance electron donation to ORR
Fluorinated Triazine COF (TP/TAPT-F) [92]	DMol3, PBE, DFT-D correction	Valence and conduction bands are primarily composed of N 2p and C 2p orbitals	4e^−^ WOR at top (N sites); 2e^−^ ORR at edges (C sites); synergistic reaction	F-doping optimizes N 2p/C 2p orbital positions for balanced bonding interactions
Tph-Dha-COF@Nb_2_C S-Scheme [93]	DMol3, PBE/DND, COSMO solvent model	E_ads_ (O_2_ at interface): −0.82 eV; E_ads_ (O_2_ at N site): −0.15 eV; ΔG (*HOOH): −1.27 eV	S-scheme charge transfer; 2e^−^ ORR at interface; IEF-driven separation	S-scheme heterojunction enhances O_2_ adsorption and reduces ORR energy barrier
TiOCs/COF-TZ (Uranium reduction) [94]	CASTEP, PBE, ultrasoft pseudopotentials	ΔG (Ti-CO_3_ coordination): −12.18 kcal/mol; LUMO shift: −2.58 to −3.80 eV	Ti^4+^-CO_3_^2−^ coordination; U(5f) electron transfer via Ti(3d) bridge	TiOCs act as electron transfer mediators without altering COF electronic structure
Tp-BTz/Tp-TTz COFs (MTG strategy) [95]	DMol3, PBE, DFT-D correction	E_ads_ (BA): Tp-BTz = −1.65 eV, Tp-TTz = −0.30 eV; ΔG_RDS_ (ORR): 0.63/0.66 eV; with BA: 0.11/0.36 eV	BA acts as proton donor; reduces PCET barrier; enhances O_2_ adsorption via polarity	BA-induced MTG reduces PCET barrier, enabling 100.9 mmol g^−1^ h^−1^ H_2_O_2_ production
BT-Py COF/g-C_3_N_4_ Heterojunction [96]	DMol3, GGA-PBE, DFT-D correction	E_ads_ (COF/CN): −2.40 eV; bandgap: CN = 2.05 eV, COF = 1.31 eV, COF/CN = 0.48 eV	Type-II heterojunction; π-π stacking; electron transfer from COF to CN	Strong interfacial π-π interactions reduce bandgap and enhance charge separation

## Data Availability

No new data were created or analyzed in this study. Data sharing is not applicable to this article.

## Abbreviations

The following abbreviations are used in this manuscript:

AIMD	Ab initio molecular dynamics
CO_2_	Carbon dioxide
COFs	Covalent organic frameworks
DFT	Density functional theory
DOS	Density of states
ESP	Electrostatic potential
*E_ad_*_s_	Adsorption energy
$E_{ext}$	Electron–nucleus interactions
$E_{H}$	Classical Coulomb interactions (Hartree energy)
$E_{xc}$	Encapsulates all many-body exchange-correlation effects
GGA	Generalized gradient approximation
Δ*G_ads_*	Adsorption free energy
H_2_O_2_	Hydrogen peroxide
LDA	Local density approximation
MOFs	Metal–organic frameworks
2D	Two-dimensional
3D	Three-dimensional
PDOS	Projected density of states
PBE	Perdew–Burke–Ernzerhof
PFASs	Polyfluoroalkyl substances
PRISMA	Preferred Reporting Items for Systematic Reviews and Meta-Analyses
$q_{\max}$	Langmuir or Sips saturation capacity
$T_{s}$	Kinetic energy of non-interacting electrons
ZPE	Zero-point energy
